# Evaluation of Cancer-Based Criteria for Use in Mainstream *BRCA1* and *BRCA2* Genetic Testing in Patients With Breast Cancer

**DOI:** 10.1001/jamanetworkopen.2019.4428

**Published:** 2019-05-24

**Authors:** Zoe Kemp, Alice Turnbull, Shawn Yost, Sheila Seal, Shazia Mahamdallie, Emma Poyastro-Pearson, Margaret Warren-Perry, Anthony Eccleston, Min-Min Tan, Soo Hwang Teo, Nicholas Turner, Ann Strydom, Angela George, Nazneen Rahman

**Affiliations:** 1 Breast Unit, Royal Marsden National Health Service Foundation Trust, London, United Kingdom; 2 Cancer Genetics Unit, Royal Marsden National Health Service Foundation Trust, London, United Kingdom; 3 Division of Genetics and Epidemiology, Institute of Cancer Research, London, United Kingdom; 4 TGLclinical, Institute of Cancer Research, London, United Kingdom; 5 DRG Abacus, Bicester, Oxfordshire, United Kingdom; 6 Cancer Research Malaysia, Subang Jaya, Selangor, Malaysia; 7 Department of Surgery, Faculty of Medicine, University Malaya, Kuala Lumpar, Malaysia

## Abstract

**Importance:**

Increasing *BRCA1* and *BRCA2* (collectively termed herein as BRCA) gene testing is required to improve cancer management and prevent BRCA-related cancers.

**Objective:**

To evaluate mainstream genetic testing using cancer-based criteria in patients with cancer.

**Design, Setting, and Participants:**

A quality improvement study and cost-effectiveness analysis of different BRCA testing selection criteria and access procedures to evaluate feasibility, acceptability, and mutation detection performance was conducted at the Royal Marsden National Health Service Foundation Trust as part of the Mainstreaming Cancer Genetics (MCG) Programme. Participants included 1184 patients with cancer who were undergoing genetic testing between September 1, 2013, and February 28, 2017.

**Main Outcomes and Measures:**

Mutation rates, quality-adjusted life-years (QALYs), and incremental cost-effectiveness ratios were the primary outcomes.

**Results:**

Of the 1184 patients (1158 women [97.8%]) meeting simple cancer-based criteria, 117 had a BRCA mutation (9.9%). The mutation rate was similar in retrospective United Kingdom (10.2% [235 of 2294]) and prospective Malaysian (9.7% [103 of 1061]) breast cancer studies. If traditional family history criteria had been used, more than 50% of the mutation-positive individuals would have been missed. Of the 117 mutation-positive individuals, 115 people (98.3%) attended their genetics appointment and cascade to relatives is underway in all appropriate families (85 of 85). Combining with the equivalent ovarian cancer study provides 5 simple cancer-based criteria for BRCA testing with a 10% mutation rate: (1) ovarian cancer; (2) breast cancer diagnosed when patients are 45 years or younger; (3) 2 primary breast cancers, both diagnosed when patients are 60 years or younger; (4) triple-negative breast cancer; and (5) male breast cancer. A sixth criterion—breast cancer plus a parent, sibling, or child with any of the other criteria—can be added to address family history. Criteria 1 through 5 are considered the MCG criteria, and criteria 1 through 6 are considered the MCGplus criteria. Testing using MCG or MCGplus criteria is cost-effective with cost-effectiveness ratios of $1330 per discounted QALYs and $1225 per discounted QALYs, respectively, and appears to lead to cancer and mortality reductions (MCG: 804 cancers, 161 deaths; MCGplus: 1020 cancers, 204 deaths per year over 50 years). Use of MCG or MCGplus criteria might allow detection of all BRCA mutations in patients with breast cancer in the United Kingdom through testing one-third of patients. Feedback questionnaires from 259 patients and 23 cancer team members (12 oncologists, 8 surgeons, and 3 nurse specialists) showed acceptability of the process with 100% of patients pleased they had genetic testing and 100% of cancer team members confident to approve patients for genetic testing. Use of MCGplus criteria also appeared to be time and resource efficient, requiring 95% fewer genetic consultations than the traditional process.

**Conclusions and Relevance:**

This study suggests that mainstream testing using simple, cancer-based criteria might be able to efficiently deliver consistent, cost-effective, patient-centered BRCA testing.

## Key Points

**Question:**

How can *BRCA1* and *BRCA2* gene testing in patients with cancer be increased?

**Findings:**

In this quality improvement study of 1184 individuals, 5 cancer-based criteria with a 10% mutation detection rate were used by the cancer team to approve genetic testing for patients with cancer.

**Meaning:**

Mainstreaming genetic testing using simple, cancer-based criteria may provide an efficient way to implement consistent, transparent, equitable, cost-effective, patient-centered genetic testing.

## Teaser

This quality improvement study and cost-effectiveness analysis evaluates mainstream genetic testing using cancer-based criteria to determine eligibility for *BRCA1* and *BRCA2* testing in patients with breast cancer.

## Introduction

Germline mutations in *BRCA1* or *BRCA2* (collectively termed herein as BRCA) contribute to approximately 12% to 15% of ovarian cancer and approximately 3% to 5% of breast cancer in most populations.^1,2,3^ Identifying BRCA mutations can deliver substantial benefits in cancer management and cancer prevention.^1,4,5^ Patients with cancer with BRCA mutations are at increased risk of a second cancer, and many choose to have bilateral mastectomy and/or salpingo-oophorectomy to mitigate this risk.^1,4^ Furthermore, specific therapeutic options for cancers driven by BRCA mutations are increasingly available.^1,5^ Identification of a BRCA mutation also provides opportunities for relatives to access improved cancer risk information, screening, and risk-reducing interventions, and it is a cost-effective cancer prevention strategy.^4,6,7,8,9,10,11^

When the BRCA genes were discovered, the time and cost of testing precluded routine integration into the cancer diagnostic pathway. Instead, family history (FH) of cancer was used to identify individuals most likely to have a BRCA mutation, with many countries using a 10% chance of a BRCA mutation as the test access threshold.^12,13,14^ However, implementation of BRCA testing has been impeded by the complexity of FH criteria and the limited FH information typically available in clinical medicine.^15,16,17^ These impediments have led to inappropriate and inconsistent testing.^18,19,20,21,22^ Furthermore, many patients with cancer with BRCA mutations are not tested because they do not fulfill FH criteria.^23,24^

These problems have led to systematic underuse and inappropriate use of BRCA testing over the past 2 decades, with consequent lost opportunities for improved cancer management and cancer prevention.^14,15,16,17,18,19,20,21,22,23,24,25,26^ A recent US study estimated that only 20% of eligible individuals are being offered testing, with more than a million eligible individuals not having testing between 2000 and 2010.^27^ It is further estimated that only 30% of patients with breast cancer and 10% of unaffected individuals with BRCA mutations in the United States have been identified.^28^ These challenges and outcomes have been similar in many other countries.^14,15,16,17,18,19,20,21,22,23,24,25,26^

To improve BRCA testing, the Mainstreaming Cancer Genetics (MCG) Programme has been developing simplified eligibility criteria and testing access processes.^29^ Ovarian cancer was addressed first, simplifying eligibility to all women with epithelial ovarian cancer, as the BRCA mutation rate is more than 10% within this group. A mainstream test access model was validated in which patients with ovarian cancer were directly approved for BRCA testing by their cancer team, with patients who were BRCA mutation-positive rather than all patients having an appointment for genetics consultation. The mainstream model has proved to be popular, efficient, and cost-effective and more than 1000 patients with ovarian cancer have had BRCA testing through the mainstream access model in the Royal Marsden National Health Service Foundation Trust.^7^ Many other centers have adopted similar processes, with comparable positive results.^30,31,32,33,34^ Herein we report on the development and evaluation of simple, cancer-based eligibility criteria for use in mainstream genetic testing in patients with breast cancer.

## Methods

### MCG-Breast

The MCG Programme was a quality improvement program aimed at making BRCA genetic testing routine for appropriate patients with cancer (eAppendix 1 in the Supplement). There were 2 subprojects: the first was led by the gynecology-oncology team and has been published^29^ and the second was the equivalent breast team project, MCG-breast. We used structured literature review, international guidelines, and real-world evidence to determine 5 simple, cancer-based criteria estimated to give an approximate 10% mutation detection rate in patients with breast cancer (eFigure 1 in the Supplement). The Royal Marsden/Institute of Cancer Research/National Institute for Health Research Specialist Biomedical Research Centre Review Board approved the study. Informed written consent was obtained from all participants; they did not receive financial compensation. This study followed the Standards for Quality Improvement Reporting Excellence (SQUIRE) reporting guideline for quality improvement studies.

### Patient Populations

The MCG-breast study ran for 3.5 years, between September 1, 2013, and February 28, 2017. A total of 1184 patients with breast cancer received genetic testing through MCG-breast during this period. Nine breast cancer predisposition genes (*BRCA1* [OMIM 113705], *BRCA2* [OMIM 600185], *PALB2* [OMIM 610355], *TP53* [OMIM 191170], *PTEN* [OMIM 601728], *STK11* [OMIM 602216], *CDH1* [OMIM 192090], *ATM* [OMIM 607585], and *CHEK2* [OMIM 604373]) were tested using the TruSight Cancer Panel (IIlumina).

While MCG-breast was running, 182 patients with breast cancer underwent testing through the genetics department because, although the proband did not fulfill an MCG-breast criterion, their FH made them eligible as they had a Manchester Scoring System (MSS) score of 15 or higher (eFigure 2 in the Supplement). These individuals are called the FH series.

We also used data from 2294 patients from the Breast and Ovarian Cancer Susceptibility (BOCS) study, a retrospective UK study of familial breast cancer, and 2575 patients from the Malaysia-Breast Cancer genetics Study (BCGS),^35^ a prospective Malaysian study of patients with cancer unselected for age or FH, to evaluate the mutation detection rates of the MCG-breast criteria. We used these patient populations to compare MCG-breast criteria with 3 widely used systems for determining BRCA testing eligibility: the MSS1,^36^ Breast and Ovarian Analysis of Disease Incidence and Cancer Estimation Algorithm (BOADICEA),^37^ and the National Comprehensive Cancer Network (NCCN) Guidelines.^38^

### Patient and Cancer Team Acceptability

We evaluated patient and cancer team acceptability of the mainstream process using similar questionnaires to the equivalent ovarian cancer programme.^29^ We initiated the patient feedback once 250 had been consented by the cancer team, sending questionnaires to 259 participants, of whom 129 replied (113 BRCA mutation-negative and 16 BRCA mutation-positive). Twenty-three members of the cancer team completed the cancer team feedback questionnaire, including 12 oncologists, 8 surgeons and 3 nurse specialists. The questionnaire and results are detailed in eTable 2 and eTable 3 in the Supplement.

### Cost-effectiveness Analyses

We performed a cost-effectiveness analysis using the model in Eccleston et al^7^ that was adapted for breast cancer. A patient-level simulation with a lifetime time horizon was constructed in Microsoft Excel (Microsoft Corp). In the model, an index population is compared for testing and no testing scenarios, patients are offered BRCA testing using mainstream testing, and cascade testing to unaffected family members is included. The analysis was conducted according to the Consolidated Health Economic Evaluation Reporting Standards (CHEERS) guideline.

The principal outcome was the cost per quality-adjusted life-year for each individual and aggregated to provide an incremental cost-effectiveness ratio. Other outcomes included total and disaggregated costs and clinical events, such as the number of new cancer cases prevented and the number of lives saved over a 50-year horizon. We also performed a probabilistic sensitivity analysis and constructed a multiple cost-effectiveness acceptability curve. Additional details on the methods are presented in eAppendix 2 in the Supplement.

## Results

### MCG-Breast

A total of 1184 individuals with breast cancer (1158 women [97.8%] and 26 men [2.2%]), underwent BRCA testing because they fulfilled 1 or more of the eligibility criteria. Of these, 117 patients had a BRCA mutation, giving an overall mutation rate of 9.9% (Table 1; eTable 1 in the Supplement). The mutation rate of each criterion exceeded 10%. Most individuals met only 1 criterion (926 [78.2%]), with 246 (20.8%) meeting 2 criteria and 12 (0.1%) meeting 3 criteria, with mutation rates of 7.7% (71 of 926), 16.3% (40 of 246), and 50.0% (6 of 12), respectively (Table 1).

**Table 1.** Cancers and BRCA Mutation Status in MCG-Breast, BOCS, and Malaysia-BCGS^a^

Cancer Status	Total Cases, No. (%)	With a BRCA Mutation, No. (%)
**MCG-Breast**		
All cases	1184	117 (9.9)
BC diagnosed when patient is ≤45 y	740 (62.5)	80 (10.8)
2 Primary BCs, both diagnosed when patient is ≤60 y	229 (19.3)	32 (14.0)
Triple-negative BC	423 (35.7)	44 (10.4)
BC+OC	36 (3.0)	10 (27.8)
Male BC	26 (2.2)	3 (11.5)
Criteria met		
1	926 (78.2)	71 (7.7)
2	246 (20.8)	40 (16.3)
3	12 (0.1)	6 (50.0)
**BOCS**		
All cases	2294	235 (10.2)
BC diagnosed when patient is ≤45 y	1630 (71.1)	181 (11.1))
2 Primary BCs, both diagnosed when patient is ≤60 y	510 (22.2)	54 (10.6)
Triple-negative BC	1073 (46.8)	127 (11.8)
Criteria met		
1	1464 (63.8)	128 (8.7)
2	741 (32.3)	87 (11.7)
3	89 (38.8)	20 (22.5)
**Malaysia-BCGS**		
All cases	1061	103 (9.7)
BC diagnosed when patient is ≤45 y	812 (76.5)	82 (10.1)
2 Primary BCs, both diagnosed when patient is ≤60 y	105 (9.9)	18 (17.1)
Triple-negative BC	321 (30.3)	41 (12.8)
BC+OC	17 (1.6)	3 (17.6)

Abbreviations: BC, breast cancer; BCGS; Breast Cancer Genetic Study; BOCS, Breast and Ovarian Cancer Susceptibility; MCG, Mainstreaming Cancer Genetics; OC, ovarian cancer.

^a^ BRCA includes *BRCA1* and *BRCA2*.

Of 117 mutation-positive individuals, 115 patients (98.3%) attended their genetics appointment. Of the 2 missing individuals, 1 declined her appointment and chemotherapy and subsequently died, and the other initially declined her appointment but then recontacted the genetics department. Cascade of information to relatives is underway in 85 families, was not relevant in 16 families, and is of unknown status in 16 families outside our catchment area. We surveyed 259 patients to evaluate acceptability of the process, of whom 129 replied (eTable 2 in the Supplement). Acceptability was high, with 100% (128 of 128) pleased that they had genetic testing and 96.1% (124 of 129) happy that testing was done by their cancer team.

The cancer team acceptability of the process was also high. All 23 team members reported feeling confident to approve patients for BRCA testing during the cancer clinic consultation and believed that the process worked well (eTable 3 in the Supplement).

### Evaluation of Testing Criteria in Other Data Sets

To further evaluate the testing eligibility criteria, we used data from 2294 participants in the BOCS Study for whom information to determine eligibility for criteria 1 through 3 was available. Of these, 235 patients (10.2%) had a BRCA mutation and each criterion also met the 10% threshold (Table 1).

To evaluate the criteria in a different population, we used data from the Malaysia-BCGS study, a prospective study of 2575 unselected women with breast cancer .^35^ Of these, 1061 patients (41.2%) were eligible for testing, of whom 103 women (9.7%) had a BRCA mutation (Table 1). Male breast cancer was not included in the study and the presence of exon deletion/duplication mutations was not tested. Consistent with the MCG-breast and BOCS data, the mutation detection rate in each category was approximately 10% (Table 1).

### Comparison With Other Eligibility Criteria

We evaluated which of the 117 mutation-positive probands would have been eligible for testing using the MSS1,^36^ BOADICEA^37^ or the NCCN criteria.^38^ Fifty-five probands (47.0%) had an MSS1 score of 15 or greater and 37 probands (31.6%) had a 10% or greater mutation probability by BOADICEA. One hundred six patients (90.6%) met the NCCN criteria, of whom 103 patients (88.0%) were eligible through their personal cancer history (eTable 1 in the Supplement).

### MCG Criteria

We combined the results of the MCG-breast and the equivalent gynecology-oncology study to develop universal testing criteria that could be used to determine eligibility of any patient with breast or ovarian cancer. We call these the MCG criteria (Figure). The MCG-breast criterion of a woman with breast and ovarian cancer became redundant because any woman with epithelial ovarian cancer has a greater than 10% chance of having a BRCA mutation.^29^ Thus, the MCG criteria are (1) ovarian cancer, (2) breast cancer diagnosed when patients are 45 years or younger, (3) 2 primary breast cancers diagnosed when patients are 60 years or younger, (4) triple-negative breast cancer, and (5) breast cancer in men (Figure).

**Figure.**
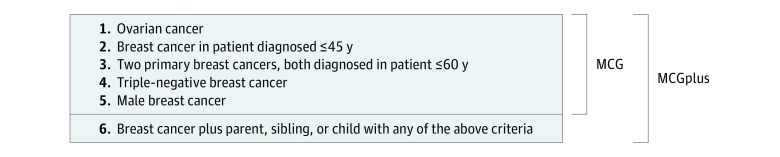
Mainstreaming Cancer Genetics (MCG) Criteria MCG includes criteria 1 through 5; MCGplus includes criteria 1 through 6. Ovarian cancer indicates epithelial ovarian cancer.

### Comparison With FH-Based Testing

During the study period, an additional 182 patients with breast cancer were tested through the genetics department. These patients did not fulfill MCG-breast criteria, but their FH made them eligible for testing because their MSS score was 15 or higher (eFigure 2 in the Supplement). Ten patients (5.5%) in this FH series had a BRCA mutation (eTable 4 in the Supplement). All 10 patients had a relative eligible for testing using the MCG criteria, and in 9 families an eligible relative was diagnosed with cancer before the proband (eTable 4 in the Supplement). A total of 151 of 182 patients (83.0%) in the FH series had an eligible relative and 86 individuals (47.3%) had an eligible first-degree relative.

The MCG-breast and FH series samples also underwent analysis of 7 additional breast cancer predisposition genes: 5 rare, higher penetrance predisposition genes (*PALB2*, *TP53*, *CDH1*, *PTEN*, and *STK11*) and 2 more common, lower penetrance predisposition genes (*ATM* and *CHEK2*).^39^ One individual (0.05%) in the FH series had a mutation in a higher penetrance non-BRCA predisposition gene compared with 24 patients (2.0%) in the MCG-breast group. In total, 9 patients (4.9%) in the FH series and 51 patients (4.3%) in the MCG-breast group had mutations in non-BRCA cancer predisposition genes (eTable 5 in the Supplement).

We next considered all 1366 individuals tested during the study period through the MCG-breast or FH series, of whom 127 patients (9.3%) had a BRCA mutation. The BRCA mutation detection rate using the different eligibility criteria are given in Table 2. The MCG and NCCN criteria performed best, with 92.1% (n = 117) and 91.3% (n = 116) mutation detection rates, respectively.

**Table 2.** Detection of BRCA Mutation by Different Eligibility Criteria^a^

Eligibility Criteria	Total No. of BRCA Mutation Carriers	BRCA Mutation Carriers Eligible for Testing, No. (%)
**All BRCA Mutation Carriers**		
MCGplus	127	123 (96.9)
MCG	127	117 (92.1)
NCCN		
All criteria	127	116 (91.3)
Personal cancer criteria	127	106 (83.5)
MSS score ≥15	127	65 (51.2)
BOADICEA ≥10	127	39 (30.7)
**BRCA Mutation Carriers Identified**		
In MCG-breast		
MCGplus	117	117 (100)
MCG	117	117 (100)
NCCN		
All criteria	117	106 (91.0)
Personal cancer criteria	117	103 (88.0)
MSS score ≥15	117	55 (47.0)
BOADICEA ≥10	117	37 (31.6)
In FH-series		
MCGplus	10	6 (60.0)
MCG	10	0
NCCN		
All criteria	10	10 (100)
Personal cancer criteria	10	0
MSS score ≥15	10	10 (100)
BOADICEA ≥10	10	2 (20.0)

Abbreviations: BOADICEA, Breast and Ovarian Analysis of Disease Incidence and Cancer Estimation Algorithm; FH, family history; MCG, Mainstreaming Cancer Genetics; MSS, Manchester Scoring System; NCCN, National Comprehensive Cancer Network.

^a^ BRCA includes *BRCA1* and *BRCA2*.

We also considered whether it were possible to extend the simple cancer-based criteria to encompass the missed mutations detected through the FH series. Six of 10 FH-series mutations (60.0%) were in patients with breast cancer with first-degree relatives who would have been eligible for testing. We therefore added a sixth category, which is breast cancer and a parent, sibling, or child (ie, a first-degree relative) with any of the other criteria. Criteria 1 through 5 are considered the MCG criteria and 1 through 6 are considered the MCGplus criteria (Figure). Applying the MCGplus criteria to the 1366 (MCG-breast plus FH series) individuals leads to an extra 86 people being eligible for testing, compared with the MCG criteria, and detection of an extra 6 mutations. Overall, MCGplus criteria detected 123 of 127 mutations (96.7%) and retained an overall mutation rate of 9.7% (123 of 1270) (Table 2).

### Cost-effectiveness

We performed a cost-effectiveness analysis using the ovarian cancer cost-effectiveness model previously described, adapted for breast cancer.^7^ We compared implementation of BRCA testing using the mainstream process and the MCG or MCGplus criteria with no testing. Both the MCG and MCGplus criteria were cost-effective with cost-effectiveness ratios of $1330 per discounted QALYs (Table 3) and $1225 per discounted QALYs (Table 4), respectively. Multiple cost-effectiveness acceptability curves showed that BRCA testing with MCG or MCGplus criteria had a greater than 99% probability of being cost-effective at a willingness-to-pay threshold of $26 184 per quality-adjusted life-year. With use of the MCG criteria, the model estimates that 804 cancers and 161 deaths would be prevented per year of testing over the subsequent 50 years. With use of the MCGplus criteria, 1020 cancers and 204 deaths are estimated to be prevented per year over 50 years.

**Table 3.** Cost-effectiveness of MCG Criteria

Outcomes	No Testing	Testing	Difference
Deaths, No.	4404	4243	−161
OC cases, No.	3047	2545	−502
BC cases, No.	989	687	−302
Total costs, $	172 525 741	175 259 610	2 733 869
Discounted QALYs	57 691	59 746	2055

Abbreviations: BC, breast cancer; MCG, Mainstreaming Cancer Genetics; OC, ovarian cancer; QALY, quality-adjusted life-year.

**Table 4.** Cost-effectiveness of MCGplus Criteria

Outcomes	No Testing	Testing	Difference
Deaths, No.	5457	5253	−204
OC cases, No.	3763	3116	−647
BC cases, No.	1202	829	−373
Total costs, $	190 223 417	193 587 091	3 363 675
Discounted QALYs	71 046	73 792	2746

Abbreviations: BC, breast cancer; MCG, Mainstreaming Cancer Genetics; OC, ovarian cancer; QALY, quality-adjusted life-year.

### Genetic Testing and Consult Requirements

Determining who is eligible for cancer predisposition gene testing based on personal history rather than FH allows one to better estimate testing and resource requirements. To exemplify this, we considered the UK outcome of implementing the mainstream testing model with the MCG or MCGplus criteria. Approximately 55 000 breast cancer cases and 7000 ovarian cancer cases are diagnosed in the United Kingdom each year. We estimate that approximately 20 000 individuals would be eligible for testing using the MCG criteria and approximately 25 000 would be eligible using the MCGplus criteria (eTable 6 in the Supplement). These numbers would lead to the identification of 2000 (MCG) or 2500 (MCGplus) BRCA mutations each year, given the mutation detection rate of approximately 10%. Approximately 12% of ovarian cancer and 3% of breast cancer diagnoses occur in BRCA mutation carriers, which is 2500 per year (840 ovarian cancers and 1650 breast cancers).^2,3^ Hence, using the MCGplus criteria theoretically allows one to identify all of the BRCA mutations in patients with breast cancer through testing of only a third of the patients. Using the mainstream access model also leads to substantial reduction in testing time and genetic consultation requirements (eFigure 3 in the Supplement). In the traditional access model, everyone has input from the genetics department before and after testing; thus, 50 000 consultations would be required if the MCGplus criteria were used. In the mainstream model, the discussions before and after the test are undertaken by the cancer team during existing appointments. The 2500 BRCA mutation-positive individuals additionally have a genetics department appointment—a 95% reduction in genetic consultation requirements. The posttest cancer and genetic management for the BRCA mutation-positive individuals and their relatives is the same in both models (eFigure 3 in the Supplement). The mainstream access model also results in approximately 85% (4 vs 25 weeks in the United Kingdom) reduction in time to test result, compared with the traditional access model (eFigure 3 in the Supplement).

## Discussion

In this study of 1184 individuals, we estimate that simple, cancer-based criteria may identify patients with breast cancer eligible for genetic testing at an approximate 10% BRCA mutation rate threshold. Our results are supported by data from 2294 patients from a retrospective UK study of familial breast cancer and 2575 patients from a prospective Malaysian study of unselected breast cancer.

Integrating these results with our equivalent study in ovarian cancer leads to 5 simple MCG criteria that can be used to determine which patients with breast or ovarian cancer should be offered genetic testing. The simplicity of the criteria makes it possible to readily and consistently determine who is eligible for testing. In turn, a mainstream testing model may be used whereby the cancer team directly performs testing instead of referring patients to the genetics department. Patients and cancer team members expressed satisfaction in this process, and it also appears to be time- and cost-efficient. In particular, mainstream testing reduces genetic consultation requirements by approximately 95%, as only the 10% mutation-positive individuals have genetic input after the test, compared with the traditional model in which all patients have genetic input before and after testing. It is recognized that, in most countries, a shortage of genetic counselors precludes offering pretest and posttest counseling to all eligible patients with cancer in a timely fashion.^40,41^ This limitation has led to ad hoc, nonvalidated testing without genetic counseling.^41^ With the cost of testing decreasing and the therapeutic implications of testing increasing, it is inevitable that testing without prior genetic counselor input will expand. Furthermore, tumor (somatic) genetic testing, which is initiated by the cancer team, is rapidly expanding and it is likely that germline and tumor genetic testing will converge over the next 5 years. We therefore believe it is preferable to explicitly institute germline genetic testing by the cancer team in close collaboration with the genetics department, as we have done herein. This cooperation ensures that the cancer team will give appropriate information before testing and that patients will receive genetic input when required. It is reassuring that we, and others, have found that patients with cancer are supportive of this testing model.^29,30,31,32,33,34^

The cancer-based criteria performed better than the FH-based MSS and BOADICEA, both of which would have missed more than 50% of the mutations. This finding is consistent with other studies showing that FH is a suboptimal selection method for identifying patients with cancer who have BRCA mutations.^23,24,29^ The mutation rate of patients in the FH series who met FH-based criteria but did not meet MCG-breast criteria was only 5.5%. This low level is to be expected; it is inevitable that the mutation rate of FH-based criteria will be reduced if one excludes patients eligible through their cancer status. Historically, such evaluations have taken the reverse approach; they have evaluated the mutation rate in patients with cancer not eligible by FH-based criteria.^12^ Given that cancer-based criteria are easier and faster to use, we believe prioritizing cancer-based criteria and adding FH-based criteria, if required, is the preferable approach. Moreover, 151 of 182 patients (83.0%) in the FH series had a relative eligible for testing by the MCG criteria, including all of the mutation-positive individuals. This finding suggests that systematic deployment of the MCG criteria would, in time, detect almost all of the mutations currently identified by FH-based criteria as well as many mutations that FH-based criteria miss. Cancer-based criteria also make it easier to estimate and evaluate testing service requirements and performance than when using FH-based criteria, as we demonstrate for the United Kingdom. Use of the cancer-based criteria should facilitate the improvement in BRCA testing delivery that many studies have shown is required.^14,15,16,17,18,19,20,21,22,23,24,25,26^

For countries without a legacy of FH-based testing or those in which FH discussions can deter testing, we believe the 5 MCG criteria offer a simple, useful way to deliver genetic testing to patients with breast and ovarian cancer. For countries that have used FH-based selection criteria for many years, this change may be too radical. To address this issue, we developed MCGplus criteria, which retain the simplicity and mutation rate of the MCG criteria and add a sixth criterion of a patient with breast cancer who has a parent, sibling, or child meeting the MCG criteria. In due course, this sixth criterion should become obsolete because the eligible relative will have been tested during their cancer diagnosis. In our center we have been using the MCGplus criteria in genetics and oncology since November 2017, and this has been well received (eFigure 4 in the Supplement).

In recent years the NCCN has expanded personal cancer-based criteria, which has led to improved mutation detection. The NCCN criteria performed similarly to the MCG criteria but are more complex, as both cancer-based and FH-based criteria are now included. This complexity makes use of the NCCN guidelines time consuming, impeding widespread adherence.^16,19,21,35^ There are strong similarities between the MCG and NCCN cancer-based criteria. A key difference is that all patients with triple-negative breast cancer are eligible by MCG criteria, but only those younger than 60 years are eligible by the NCCN. Ten of 44 mutation-positive women (22.7%) with triple-negative breast cancer in MCG-breast were diagnosed when they were older than 60 years. Moreover, the mutation rate in triple-negative breast cancer at any age in the MCG-breast, BOCS, and Malaysia-BCGS data was greater than 10%, and this finding is consistent with other studies.^12,42,43^ It is noteworthy that 106 of 116 mutations (91.4%) detected by the NCCN criteria were in patients eligible for testing due solely to their cancer history, although many also fulfilled FH criteria. This finding suggests that there may be redundancy in the NCCN cancer-based and FH-based criteria that could be streamlined.

Using MCG or MCGplus criteria is cost-effective and appears to result in meaningful cancer and mortality reductions. BRCA testing using many different criteria and testing processes has been shown to be cost-effective.^4,6,7,8,9,10,11^ The combined economic benefit and increasing clinical utility of testing appear to provide a strong argument for relaxing the mutation threshold and expanding testing to more patients with breast cancer. Changing the age threshold for breast cancer to 50 years or younger or 60 years or younger and removing MCG criterion 2 would be the logical expansions and simplifications of the MCG criteria. Ultimately, offering testing to all patients with breast cancer will likely occur. However, currently, most centers have limited resources they must use for testing at a specified mutation detection threshold.

### Limitations

The study has limitations. The cost-effectiveness analysis we performed compared testing with no testing. When we initiated the study, no germline genetic testing in oncology was performed in the United Kingdom, so testing vs no testing was considered the most appropriate comparison. Furthermore, a major aspiration of the MCG Programme was to provide data and resources that could be used by the many centers and countries that do not yet offer BRCA testing. However, for some centers, for example, those with surplus genetic resources that can be used in oncology, a more appropriate comparison would be between the MCG criteria and FH-based criteria.

Concurrent testing of other cancer predisposition genes in addition to the BRCA genes is being increasingly performed.^44^ We suggest that the mainstream testing process can be readily adapted for multigene testing; in our study, it led to an extra 51 (40% increase) posttest genetic consultations for patients with mutations in non-BRCA genes. However, we did not evaluate the performance of MCG criteria for non-BRCA genes. We believe that MCG criteria are likely to perform at least as well as other criteria because more eligible patients receive testing using the MCG criteria and mainstream testing process.^45,46^ The MCG criteria also do not address BRCA testing in patients with cancers other than breast or ovarian. To our knowledge, there is no evidence that personal history of cancers other than breast or ovarian would meet a 10% mutation detection threshold.

## Conclusions

It is well known that underuse and inappropriate use of genetic testing in patients with cancer is impeding cancer management and prevention.^14,15,16,17,18,19,20,21,22,23,24,25,26^ Herein, we presented a streamlined genetic testing process based on short, simple eligibility criteria that can be used by the cancer team. We suggest that mainstreaming testing using these criteria may be an efficient way to deploy limited resources in a consistent, transparent, equitable, cost-effective, patient-centered manner.

## Notes

Supplement. **eAppendix 1.** MCG Programme Protocols and Resources **eAppendix 2.** Methods **eReferences** **eTable 1.** BRCA Mutation-Positive Individuals Identified in the MCG-breast study **eTable 2.** Summary of Patient Feedback **eTable 3.** Summary of Cancer Team Feedback **eTable 4.** Eligibility and Family History of BRCA Mutation-Positive Individuals in FH-Series **eTable 5.** Mutations in Non-BRCA Breast Cancer Predisposition Genes **eTable 6.** Annual BRCA Tests Required in UK Using MCG or MCGplus criteria **eFigure 1.** MCG-breast Process and Patient Flow Timeline **eFigure 2.** BRCA Testing Protocol Used in Genetics Prior to MCG-breast **eFigure 3.** Impact on Testing and Genetic Consultations Requirements of MCGplus in UK **eFigure 4.** BRCA Testing Protocol Used in Oncology and Genetics From 11/2017
