# Supplementary material for: Evaluation of Cancer-Based Criteria for Use in Mainstream BRCA1 and BRCA2 Genetic Testing in Patients With Breast Cancer
Source: JAMA Netw Open. 2019 May 24;2(5):e194428. doi: 10.1001/jamanetworkopen.2019.4428 (PMC6632150; doi:10.1001/jamanetworkopen.2019.4428)
Supplement: Supplement. — eAppendix 1. MCG Programme Protocols and Resources eAppendix 2. Methods eReferences eTable 1. BRCA Mutation-Positive Individuals Identified in the MCG-breast study eTable 2. Summary of Patient Feedback eTable 3. Summary of Cancer Team Feedback eTable 4. Eligibility and Family History of BRCA Mutation-Positive Individuals in FH-Series eTable 5. Mutations in Non-BRCA Breast Cancer Predisposition Genes eTable 6. Annual BRCA Tests Required in UK Using MCG or MCGplus criteria eFigure 1. MCG-breast Process and Patient Flow Timeline eFigure 2. BRCA Testing Protocol Used in Genetics Prior to MCG-breast eFigure 3. Impact on Testing and Genetic Consultations Requirements of MCGplus in UK eFigure 4. BRCA Testing Protocol Used in Oncology and Genetics From 11/2017 [file jamanetwopen-2-e194428-s001.pdf]

Supplementary Online Content

Kemp Z, Turnbull A, Yost S, et al. Evaluation of cancer-based criteria for use in mainstream *BRCA1* and *BRCA2* genetic testing in patients with breast cancer. *JAMA Netw Open*. 2019;2(5):e194428. doi:10.1001/jamanetworkopen.2019.4428

eAppendix 1. MCG Programme Protocols and Resources

eAppendix 2. Methods

eReferences

eTable 1. BRCA Mutation-Positive Individuals Identified in the MCG-breast study

eTable 2. Summary of Patient Feedback

eTable 3. Summary of Cancer Team Feedback

eTable 4. Eligibility and Family History of BRCA Mutation-Positive Individuals in FH-Series

eTable 5. Mutations in Non-BRCA Breast Cancer Predisposition Genes

eTable 6. Annual BRCA Tests Required in UK Using MCG or MCGplus criteria

eFigure 1. MCG-breast Process and Patient Flow Timeline

eFigure 2. BRCA Testing Protocol Used in Genetics Prior to MCG-breast

eFigure 3. Impact on Testing and Genetic Consultations Requirements of MCGplus in UK

eFigure 4. BRCA Testing Protocol Used in Oncology and Genetics From 11/2017

This supplementary material has been provided by the authors to give readers additional information about their work.

© 2019 Kemp Z et al. *JAMA Network Open*.

eAppendix 1. MCG Programme protocols and resources

Mainstreaming Cancer Genetics (MCG) Programme

BRCA Testing Implementation Resources

The following pack contains the standard resources used by the MCG for implementation of mainstream BRCA testing and a Frequently Asked Questions document.

Protocols are provided for the MCG-breast study, MCG BRCA testing eligibility criteria and the MCGplus BRCA testing eligibility criteria. MCG and MCGplus include 5 criteria based on cancer patient characteristics. MCGplus has a 6th family history category.

All have a mutation detection rate of ~10%.

The following are included:

- MCG-breast testing protocol
- MCG BRCA testing protocol
- MCGplus BRCA testing protocol
- MCG F1 Consent form for genetic testing
- MCG IS1 BRCA gene testing information sheet
- MCG IS2 Normal BRCA result information sheet
- MCG IS3 BRCA mutation result information sheet
- MCG IS4 BRCA VRE result information sheet
- MCG Implementation FAQs

MCG-breast testing protocol

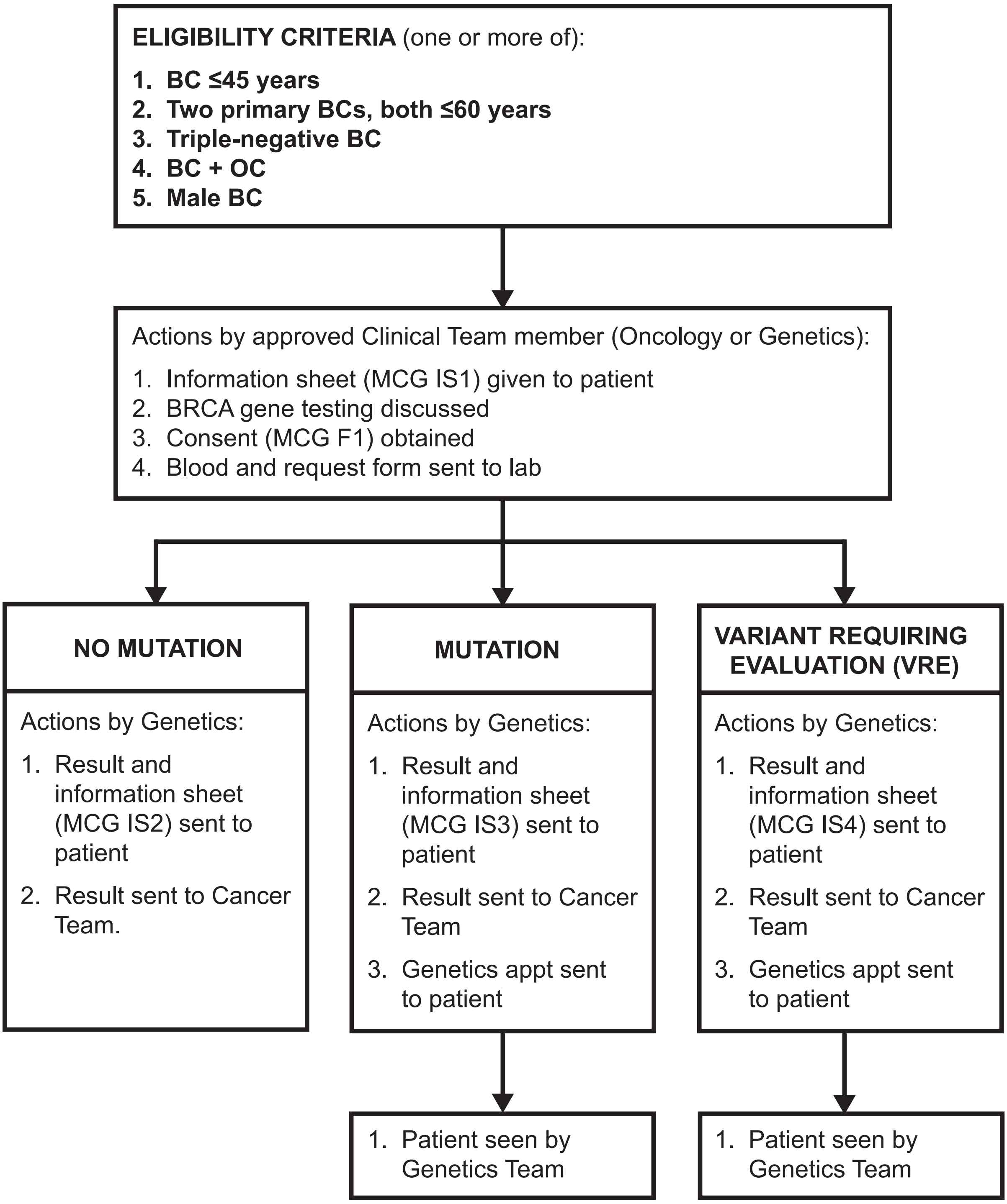

© 2019 Kemp Z et al. *JAMA Network Open*.

BRCA testing protocol - MCG criteria

MCG BRCA testing eligibility criteria

Individual with any of the following:

1) Ovarian cancer
2) Breast cancer ≤45 years
3) Two primary breast cancers, both ≤60 years
4) Triple-negative breast cancer
5) Male breast cancer

Process

Actions by Oncology or Genetics:

1. Information sheet (MCG IS1) given to patient.
2. BRCA gene testing discussed.
3. Consent (MCG F1) obtained.
4. Blood and test request form sent to lab.

NO MUTATION

Actions by Genetics:

1. Result and information sheet (MCG IS2) sent to patient.
2. Result sent to Cancer Team.

MUTATION

Actions by Genetics:

1. Result and information sheet (MCG IS3) sent to patient.
2. Result sent to Cancer Team
3. Genetics appt sent to patient.

VARIANT REQUIRING EVALUATION (VRE)

Actions by Genetics:

1. Result and information sheet (MCG IS4) sent to patient.
2. Result sent to Cancer Team.
3. Genetics appt sent to patient.

© 2019 Kemp Z et al. *JAMA Network Open.*

BRCA testing protocol - MCGplus criteria

MCGplus BRCA testing eligibility criteria

Individual with any of the following:

1) Ovarian cancer
2) Breast cancer ≤45 years
3) Two primary breast cancers, both ≤60 years
4) Triple-negative breast cancer
5) Male breast cancer
6) Breast cancer + parent, child or sibling with any of the above criteria

Process

Actions by Oncology or Genetics:

1. Information sheet (MCG IS1) given to patient.
2. BRCA gene testing discussed.
3. Consent (MCG F1) obtained.
4. Blood and test request form sent to lab.

NO MUTATION

Actions by Genetics:

1. Result and information sheet (MCG IS2) sent to patient.
2. Result sent to Cancer Team.

MUTATION

Actions by Genetics:

1. Result and information sheet (MCG IS3) sent to patient.
2. Result sent to Cancer Team
3. Genetics appt sent to patient.

VARIANT REQUIRING EVALUATION (VRE)

Actions by Genetics:

1. Result and information sheet (MCG IS4) sent to patient.
2. Result sent to Cancer Team.
3. Genetics appt sent to patient.

© 2019 Kemp Z et al. *JAMA Network Open.*

Consent for Genetic Testing

**XXX HOSPITAL NAME XXX**<br><br>**Patient / parental agreement to investigation**	Patient's surname/family name
	Patient's first names
	Date of birth
	Responsible health professional
	Job title
	Hospital number (or other identifier)
**See overleaf for:**<br>**(1) Special Requirements (2) Information provided and (3) Consent policy**	☐ Male ☐ Female

Statement of health professional (to be filled in by health professional with appropriate knowledge of proposed procedure, as specified in consent policy)

The purpose of these investigations is to help establish the causes of cancer and/or risk of cancer for you. The results may also provide information which may be helpful for other family members. We have provided written information outlining the risks and benefits of these investigations. During the consultation and/or through written information we have discussed the following issues related to gene testing and you have agreed to the numbered statements indicated below. If you have further questions please contact the genetics team on **XXX**

Please circle as appropriate

1	I agree to the testing of	Yes		
2	I understand that the test results will be put on the electronic patient records	Yes		
3	I understand the sample will be stored in case new gene tests become available.	Yes		
4	I understand that additional tests may be undertaken on the stored sample, if indicated, and I will be informed of any relevant results.	Yes		
5	I agree that the test results can be made available to doctors looking after family members, on request.	Yes	No	N/A
6	I agree to the sample being used anonymously for research.	Yes	No	

Additional issues discussed:

Signature: ____________________________ Print name: ___________________________ Date: ______________
(Patient / Parent / Guardian / Relative)

To be completed if individual identified above is deceased:
I agree to the above genetic tests and sharing of information on behalf of my relative.
Relationship to patient: __________________________________

Signature: ____________________________ Print name: ___________________________ Date: ______________
(Clinician)

To be retained in patient's notes

© 2019 Kemp Z et al. *JAMA Network Open*.

The following information leaflet / consultation letter(s) have been provided:

☐ ……………………………………………………………… …………………. (version no._________)

☐ ……………………………………………………………………………………… (version no._________)

Special requirements (e.g. other language/other communication method):

………..

………..

Guidance to health professionals (to be read in conjunction with consent policy)

This form

This form documents the patient's agreement (or that of a person with parental responsibility for the patient) to go ahead with the investigation you have proposed**. It is only designed for use where the patient is expected to remain alert throughout and where an anaesthetist is not involved in their care.**

Consent forms are not legal waivers – if patients, for example, do not receive enough information on which to base their decision, then the consent may not be valid, even though the form has been signed. Patients also have every right to change their mind after signing the form.

Who can give consent

Everyone aged 16 or more is presumed to be competent to give consent for themselves, unless the opposite is demonstrated. If a child under the age of 16 has "sufficient understanding and intelligence to enable him or her to understand fully what is proposed", then he or she will be competent to give consent for himself or herself. Young people aged 16 and 17, and legally 'competent' younger children, may therefore sign this form for themselves, if they wish. If the child is not able to give consent for himself or herself, some-one with parental responsibility may do so on their behalf. Even where a child is able to give consent for himself or herself, you should always involve those with parental responsibility in the child's care, unless the child specifically asks you not to do so. If a patient is mentally competent to give consent but is physically unable to sign a form, you should complete this form as usual, and ask an independent witness to confirm that the patient has given consent orally or non-verbally.

When NOT to use this form (see also 'This form' above)

If the patient is 18 or over and lacks the capacity to give consent, you should use form 4 (form for adults who lack the capacity to consent to investigation or treatment) instead of this form. A patient lacks capacity if they have an impairment of the mind or brain or disturbance affecting the way their mind or brain works and they cannot:

- understand information about the decision to be made
- retain that information in their mind
- use or weigh that information as part of the decision-making process, or
- communicate their decision (by talking, using sign language or any other means).

You should always take all reasonable steps (for example involving more specialist colleagues) to support a patient in making their own decision, before concluding that they are unable to do so.
Relatives **cannot** be asked to sign a form on behalf of an adult who lacks capacity to consent for themselves, unless they have been given the authority to so under a Lasting Power of Attorney or as a court appointed deputy.

Information

Information about what the investigation will involve, its benefits and risks and the alternatives to the particular test proposed, is crucial for patients when making up their minds about investigations. The courts have stated that patients should be told about 'significant risks which would affect the judgement of a reasonable patient'. 'Significant' has not been legally defined, but the GMC requires doctors to tell patients about 'serious or frequently occurring' risks. In addition if patients make clear they have particular concerns about certain kinds of risk, you should make sure they are informed about these risks, even if they are very small or rare. You should always answer questions honestly. Sometimes, patients may make it clear that they do not want to have any information about the options, but want you to decide on their behalf. In such circumstances, you should do your best to ensure that the patient receives at least very basic information about what is proposed. Where information is refused, you should document this overleaf or in the patient's notes.

The law on consent

See the Department of Health's *Reference guide to consent for examination or treatment* for a comprehensive summary of the law on consent (also available at www.dh.gov.uk/consent).

To be retained in patient's notes

© 2019 Kemp Z et al. *JAMA Network Open.*

BRCA1 and BRCA2 gene testing

Information sheet for patients with cancer

In most people cancer occurs by chance. In a minority of people with ovarian cancer (about 15%) or breast cancer (about 3%), cancer occurs because they have a mutation in the BRCA1 or BRCA2 gene.

BRCA1 and BRCA2 mutations result in increased risks of breast and ovarian cancer. They occur more frequently in women who have both breast and ovarian cancer, those with particular types of cancer, and if there is a strong family history of breast and/or ovarian cancer. It is important to identify if a cancer is due to a BRCA1 or BRCA2 mutation because it provides you and your doctors with information that can help treat your cancer and to reduce your risk of future cancer. It can also provide information for relatives about their risks of cancer.

Why am I being offered this test?
You are being offered a test to look for mutations in BRCA1 and BRCA2 because of your cancer diagnosis.

What are the benefits to me?
Knowing whether or not you carry a mutation in BRCA1 or BRCA2 gives the cancer team more information about your cancer. This can help decisions about the treatments they recommend for you, for example which chemotherapy drugs or surgery would be most suitable. It will also give better information about your risk of developing cancer in the future.

Does having the test have implications for my family?
In most people the test will be normal and we will not find a gene mutation. This would be reassuring for relatives as it would indicate that your cancer was unlikely to be due to hereditary factors that would put them at very high risk of cancer. If your test shows you have a gene mutation, it is possible that some relatives also have the mutation. Relatives would be able to discuss this with a specialist geneticist and have a test if they chose to.

What will happen if NO mutation in BRCA1 or BRCA2 is found?
This is the most likely outcome, as most women with cancer do not have a mutation in BRCA1 or BRCA2. This would be reassuring in suggesting you are unlikely to be at high risk of developing another, new cancer in the future. The cancer team will be able to use this information in their management decisions. Very occasionally mutations in other genes can be involved in causing breast or ovarian cancer. Also new discoveries are being made all the time. If a new gene test becomes available in the future the genetics team may be able to do the test using the sample you have already provided and would send the result to you and the cancer team. If your doctors think other genetic factors might be involved in your cancer they can ask the genetics clinic to send you an appointment to evaluate this, if you have not already had an appointment with genetics.

© 2019 Kemp Z et al. *JAMA Network Open.*

What will happen if a BRCA1 or BRCA2 mutation is found?
Your cancer team will use the information in their management decisions. The genetics team will send you an appointment to discuss the results and address any questions you have. They will also discuss what the test result means for your future risk of cancer, your options for future screening and measures to reduce these risks. They will evaluate your family history and can provide information for the appropriate family members should they wish to consider testing to see if they have inherited the mutation. Any relatives can be referred to a Genetics Unit to discuss this further.

What will happen if the test result is unclear?
Very occasionally (<1%) we find a gene change, known as a 'variant', that needs further assessment before we can decide if it is linked to why you have had cancer. If this occurs, the genetics team will send you an appointment to explain the result and to discuss with you what further information and/or tests would be helpful to find out if the variant is linked to your cancer.

Do I have to have the test?
No, having this test is optional. Your decision will not affect the standard of care you receive from the hospital or doctor, which will be based on the available information.

What if I am not sure if I want to have the test?
We would recommend for you to have further discussions with a specialist member of the genetics team.

What will happen next if I say yes?
If you decide to have the test, you will be asked to sign a consent form. A blood sample will be taken for the test.

How will I receive the results of the test?
The genetics team will send you and your cancer team the results of the test by post. The results may take up to 4 weeks, but will usually be within 3 weeks.

Will my information be confidential?
All data collected about you will be held under the provisions of the 1998 Data Protection Act and stored in secure files. The only people who will know your identity are the hospital staff and a few trained staff reporting the results who are bound by a professional duty to protect your privacy.

If you have any questions please contact XXX on XXX

© 2019 Kemp Z et al. *JAMA Network Open.*

Receiving a normal BRCA1 and BRCA2 test result

Information sheet for patients with cancer

You had a BRCA1 and BRCA2 gene test because you have had cancer.

The test result is normal.
No BRCA1 or BRCA2 mutation (gene change) was identified in your blood sample.

What does this result mean for me?
This means we have not found a BRCA1 or BRCA2 mutation which would put you at high risk of developing another cancer. The cancer team will discuss if this normal result has any implications for your cancer management.

A normal result is common. In most women with breast and/or ovarian cancer no mutation in BRCA1 or BRCA2 is found. If you have a strong family history of breast and/or ovarian cancer, or a strong family history of other cancers, or if you developed cancer at an unusually young age, it may be helpful to look into things further. The genetics or cancer team will discuss this with you, if appropriate.

Very occasionally mutations in other genes can be involved in causing breast or ovarian cancer. Also new discoveries are being made all the time. If a new gene test becomes available the genetics team may be able to do the test using the sample you have already provided and they will send the result to you and your cancer team.

What does this result mean for my relatives?
This result is good news for your relatives, as it means they are unlikely to be at high increased risk of developing breast and/or ovarian cancer themselves. You may wish to share this result with them.

All women are eligible to have mammograms from 47 years in the National Breast Screening Programme. Depending on the family history, some women may be eligible for mammograms from 40 years, even if there has been a normal BRCA1 or BRCA2 gene test in the family. There is currently no known effective form of ovarian screening. If a woman has multiple relatives with ovarian cancer removal of the ovaries is sometimes considered.

If any of your relatives wish to discuss their own risks of cancer further they should speak with their GP who can refer them for further discussions at a Family History or Genetics clinic.

If you have any further questions, please contact XXX on XXX

© 2019 Kemp Z et al. *JAMA Network Open.*

Receiving a BRCA1 and BRCA2 test result that identifies a mutation

Information sheet for patients with cancer

You had a BRCA1 and BRCA2 gene test because you have had cancer.

The test result has shown that you have a mutation (gene change) in either the BRCA1 or BRCA2 gene. The exact details of the mutation are given in the test report.

BRCA1 or BRCA2 mutations result in increased risks of breast and ovarian cancer, and occasionally other cancers. Therefore this result provides an explanation for why you developed cancer.

Your cancer team will discuss with you if this result has implications for your cancer treatment and/or follow-up.

This result has implications for your future health and potentially for your relatives. An appointment has been made for you in the Genetics clinic to discuss these issues further. At the appointment you will be able discuss your future risks of cancer and your options for cancer screening and measures to reduce the risk of cancer. The potential implications for relatives will also be discussed. The processes by which your relatives can have discussions themselves to decide if they wish to have testing will be explained.

You may find it helpful to read the information booklet "A Beginner's Guide to BRCA1 and BRCA2" which gives more detailed information. This can be downloaded from www.royalmarsden.nhs.uk/brca

If you need to discuss anything urgently prior to your appointment, or wish to alter the date of your appointment, please contact XXX on XXX

© 2019 Kemp Z et al. *JAMA Network Open*.

Receiving a BRCA1 and BRCA2 test result that identifies a variant requiring evaluation (VRE)

Information sheet for patients with cancer

You had a BRCA1 and BRCA2 gene test because you have had cancer.

The test result has shown that you have a gene change (variant) in either the BRCA1 or BRCA2 gene that requires further evaluation.

At the moment, we do not have enough information to decide if this variant is linked to why you have had cancer.

Variants in the BRCA1 and BRCA2 genes are common, and most do not cause cancer. Very occasionally, we find a variant that requires further assessment before we can decide if it leads to an increased risk of cancer. In some cases, we may need to do further blood tests to help us find out more about the impact of the variant.

We have made an appointment for you in the Genetics clinic to discuss your result further. At the appointment we will explain in more detail about the result and any further tests that may be required. We will also discuss the process and timeframe for deciding if the variant is likely to be linked to your cancer.

It is important for us to have as much information as possible when we see you. We have enclosed a family history questionnaire with your letter, and would be very grateful if you would fill this in and return it to us before we see you in clinic.

If you need to discuss anything urgently prior to your appointment, or wish to alter the date of your appointment, please contact XXX on XXX

© 2019 Kemp Z et al. *JAMA Network Open.*

MCG BRCA testing protocol implementation
Frequently asked questions

Last updated: 01/08/2018

Q: What are the MCG eligibility criteria for BRCA1 and BRCA2 mutation (termed BRCA) testing?
The MCG eligibility for BRCA testing (i.e. to look in blood for mutations in the BRCA1 and BRCA2 genes) in breast cancer (BC) or ovarian cancer (OC) patients are as follows:

1) Ovarian cancer
2) Breast cancer ≤45 years
3) Two primary breast cancers, both ≤60 yrs
4) Triple-negative breast cancer
5) Male breast cancer

Q: What are the MCGplus eligibility criteria for BRCA mutation testing?
The MCGplus eligibility criteria for BRCA testing are the same as the MCG criteria but include an additional criterion as follows:

1) Ovarian cancer
2) Breast cancer ≤45 years
3) Two primary breast cancers, both ≤60 yrs
4) Triple-negative breast cancer
5) Male breast cancer
6) Breast cancer and a parent, child or sibling with any of the above criteria

Q: How were the eligibility criteria for BRCA testing decided?
The eligibility criteria for BRCA testing are in line with UK NICE recommendations www.nice.org.uk/guidance/cg164 which state that any patient with ≥10% chance of having a BRCA mutation should be tested. Similar guidelines exist globally. Extensive evaluation and data audit has shown that patients meeting the above eligibility criteria are at ≥10% risk of a BRCA mutation.

Q: Are the eligibility criteria the same in Oncology and Genetics?
Yes. The same eligibility criteria are used for patients tested through Oncology and Genetics Units.

Q: Are all patients with ovarian cancer eligible for BRCA testing?
Patients with epithelial ovarian cancer are eligible for BRCA testing. Epithelial ovarian cancer is cancer which started in the surface layer covering the ovary. It is the most common type of ovarian cancer accounting for >90% cases.

Q: What is triple-negative breast cancer?
Triple-negative breast cancer is a breast cancer negative for oestrogen receptor (ER), progesterone receptor (PR) and HER2 expression. For the purpose of genetic testing this means an Allred score of 0, 1 or 2 for ER and PR receptors and HER2 Score of 0 or 1+ by immunohistochemistry, or a score of 2+ by immunohistochemistry and DDISH negative

© 2019 Kemp Z et al. *JAMA Network Open*.

Q: Who can perform BRCA testing in patients?
Geneticists and non-geneticists can perform BRCA testing. Non-geneticists should feel competent and confident to perform testing. MCG developed a short (~20) online training for non-geneticists which is available at www.mcgprogramme.com.

Q: When should discussion of BRCA testing be undertaken?
This should be at the discretion of the clinician. BRCA testing can be discussed and undertaken at the time of diagnosis, during active cancer management or during follow up. However, if the result is required for management decisions, timing of testing must be planned accordingly.

Q: What information should I give to the patient prior to obtaining consent?
An information sheet such as the 'BRCA1 and BRCA2 gene testing - Information sheet for patients with cancer' (MCG IS1) should be given to the patient. Patients should be informed that BRCA mutations are a cause of cancer and knowing whether or not a BRCA mutation is involved in causing their cancer can be helpful for their current and future management. The clinician may like to describe the specific relevance of the test for the specific patient. The patient should also be aware that the result can provide information of relevance to the wider family. However, it is important to remember that most tests are normal and therefore detailed discussions regarding risk management for patient and relatives, prior to testing, are not required. If a patient has questions that require either more time or more expertise than you are able to provide, the patient should be referred to Genetics. The training provides further information about BRCA testing.

Q: What should I do if a patient has additional queries before BRCA testing?
Patients with additional queries can be referred to Genetics for an appointment and further discussion. Contact details for Genetics should be readily available.

Q: Which consent form should I use?
The 'Consent for Genetic Testing' form (MCG F1) used by MCG is available at www.mcgprogramme.com. The training provides further information about how to take consent.

Q: How long does it take to get a BRCA result?
The results of full analysis of the BRCA1 and BRCA2 genes typically take about ~3 weeks, but may take longer. You should check with your local laboratory.

Q: Should *in situ* breast cancer be included when assessing eligibility?
Yes. *In situ* cancer, such as DCIS (ductal carcinoma in situ) and LCIS (lobular carcinoma in situ), should be included in the same way as invasive breast cancer in assessing eligibility for BRCA testing.

Q: How should multiple metachronous ipsilateral breast cancers be assessed?
Two (or more) separate, ipsilateral breast cancers which have occurred 5 or more years apart should be considered as separate cancers in the assessment of eligibility for BRCA testing (i.e. they should be counted as a bilateral breast cancer), unless it is clear the second cancer is a recurrence. This is a pragmatic approach as it is currently not possible to robustly identify which are separate primaries and which recurrence, but most are likely to be separate cancers.

Q: How should multiple synchronous ipsilateral breast cancers be assessed?
These should be counted as a single breast cancer for assessing eligibility for genetic testing. Simultaneous ipsilateral breast cancers are sometimes termed multifocal or multicentric.

Q: How strict are the age cut-offs for testing?
Age cut-offs are strictly applied. For example, a woman with bilateral breast cancer diagnosed at 58 years and 61 years would not be eligible for testing, but if she were diagnosed at 58 years and 60 years she would be eligible. It is recognised, and inevitable, that individuals close to a threshold may have similar likelihoods of carrying a mutation but different eligibility. We are working hard to

© 2019 Kemp Z et al. *JAMA Network Open.*

make eligibility generally more permissive, but in the meantime it is important for clinicians and patients to have confidence that criteria are being consistently applied.

Q: Can BRCA testing be undertaken in individuals who do not meet eligibility criteria?
Yes. Patients who do not meet any of the eligibility criteria can have a self-funded test. It should be made fully clear to these patients that the chance of detecting a mutation is <10%. Self-funding patients should be consented in the normal way.

Q: What if a patient meets the criteria but chooses not to have a test?
The test is optional. A patient may decline to be tested, ask to have longer to think about testing or be referred to Genetics if they want, or need, more detailed discussions.

Q: What if a family member has already had BRCA testing and a mutation was found?
If a member of the family has already had a BRCA test and a mutation was found this should be noted on the lab form. It may influence the testing that is performed.

Q: What if a relative has already been tested and does not carry a BRCA mutation?
A BRCA test can still be performed on a second individual within the family if they meet the eligibility criteria.

Q: Are unaffected individuals eligible for BRCA testing?
Unaffected individuals with a family history of cancer are not eligible for NHS-funded BRCA testing in our centre. Recent data has shown that the mutation rate is well below the 10% NICE threshold for testing. Testing should be performed in an eligible cancer patient in the family if possible. Or the individual can have a self-funded test.

Q: Who gives the patient the result of the BRCA test?
In our centre the Genetics team writes to the patient with the result and send an information sheet with additional information. The referring clinician and GP are also notified. The result is uploaded to the electronic patient record.

Q: What happens if no mutation is identified?
The Genetics team inform the patient of the result in writing and send the patient a copy of the report and the information sheet 'Receiving a normal BRCA1 and BRCA2 test result' (MCG IS2). The Cancer team should use the information as appropriate for their cancer management. Usually no further input is required from Genetics. If the patient has an unusual cancer history or extensive family history of cancer or has questions about the result, an appointment should be arranged with the Genetics team.

Q: What happens if a mutation is identified?
The Genetics team inform the patient in writing and send the patient a copy of the report and the information sheet 'Receiving a BRCA1 and BRCA2 test result that identifies a mutation' (MCG IS3) and an appointment for the Genetics clinic. The Cancer team should use the information as appropriate for their cancer management. The Genetics team will discuss with the patient the implications for their future cancer risk and will also evaluate which relatives may be impacted. The processes for cascading the information to relatives will be explained.

Q: What if there is a variant requiring evaluation (VRE) identified?
Very occasionally (<1%), we identify a variant that does not fulfil the criteria for pathogenic mutations, but requires further evaluation. In such cases, an information sheet (MCG IS4) and an appointment with Genetics is sent to the patient. The result and further analyses required are discussed with the patient. Once the additional evaluation has been completed (typically 2-6 months) the patient and clinician are informed of the final management class. Variants are only classified as VREs if there is suggestive evidence of pathogenicity that can potentially be confirmed by additional analyses (e.g. a splicing assay).

© 2019 Kemp Z et al. *JAMA Network Open.*

Q: What if new evidence in the future shows a variant is pathogenic?
We keep all variants identified under review and if any are reclassified Genetics will automatically re-issue reports and clear, revised recommendations. It is important to remember that rare variants in these genes are collectively common in the general population (present in about 10%), and the great majority are not pathogenic.

Q: If a mutation is identified who will follow-up the patient's relatives?
The Genetics team give the patient a "To whom it may concern letter" to give to relatives. The letter explains that a cancer predisposition gene mutation has been identified in the family and that relatives can ask their GP to refer them to a genetics service to discuss the implications. This is standard practice in Genetics in UK.

Q: If the patient does not have a BRCA mutation, are there additional genetic tests that should be performed?
Panel testing of multiple cancer genes is now performed in many centres. Otherwise some patients may be eligible for further tests, particularly if they were diagnosed at a particularly young age, if they have multiple primary cancers or if there is an extensive family history of cancer. We recommend that such patients are referred to Genetics.

Q: What are the insurance implications for cancer patients?
In the UK if a cancer patient applies for life cover, critical illness or income protection cover after the gene test is performed then it will need to be disclosed, along with the other information about their cancer diagnosis. This is unlikely to have impact on the cover they are offered over and above the impact of their cancer diagnosis. If the gene test was performed after an insurance policy was set-up the result does NOT need to be disclosed.

Q: Are there insurance implications for the cancer patients relatives?
In the UK relatives need to tell the insurance company about the cancer diagnosis and if a gene mutation has been found when asked about their family history (if they are aware of it). If the test is normal some insurance companies may take this into consideration to mitigate the unfavourable impact of the family history. Unaffected individuals do not have to disclose the results of predictive gene testing to insurance companies but may choose to do so, particularly if the test is negative.

Q. How cost-effective is BRCA genetic testing using the MCG and MCGplus criteria?
Genetic testing using the MCG and MCGplus criteria is highly cost-effective as documented in our papers.

© 2019 Kemp Z et al. *JAMA Network Open.*

eAppendix 2. Methods

MCG-breast

MCG-breast was a quality improvement programme designed to evaluate use for breast cancer of the mainstream genetic testing process developed and validated for ovarian cancer as part of the Mainstreaming Cancer Genetics (MCG) programme (www.mcgprogramme.com).[1] Detailed information about the MCG programme resources is given in eAppendix 1. The core of the mainstream access model is that eligible cancer patients can be consented to genetic testing by cancer team members who have completed a short online training module.[1] In the UK, and many other countries, it is recommended that individuals at ≥10% chance of a BRCA mutation should be offered testing.[2] We used structured literature review, international guidelines and real world evidence to determine five, simple cancer-based criteria we estimated would give ~10% mutation detection rate (Supplement eFigure 1). Initially criterion one was breast cancer (BC) ≤40, consistent with National Comprehensive Cancer Network (NCCN) guidelines. Whilst MCG-breast was running NCCN changed their eligibility to BC ≤45 years.[3] We similarly altered the MCG-breast criterion. We then used the electronic patient record to identify breast cancer patients between 41-45 years seen in the cancer clinic since MCG-breast began and they were offered BRCA testing. We define two primary breast cancers as bilateral cancers or two ipsilateral cancers which have occurred more than five years apart. We define ovarian cancer as epithelial ovarian cancer, which constitutes ~90% of cases. We define triple-negative breast cancer (TNBC), as a cancer with an Allred score of 0 or 2 for estrogen receptor (ER) and progesterone receptor (PR) and a human epidermal growth factor 2 (HER2) score of 0 or 1+ by immunohistochemistry, or 2+ by immunohistochemistry and dual-colour, dual-hapten brightfield in situ hybridisation (DDISH) negative.

The Royal Marsden/ICR NIHR Specialist Biomedical Research Centre review board approved the study and determined it was not human subjects research requiring separate consent because genetic testing in patients at 10% chance of having a BRCA mutation was existing practice. Informed written consent was therefore obtained from all participants using the standard genetic test consent form (eAppendix 2).

The 3.5 year programme ran in the Royal Marsden NHS Foundation Trust from Sept 2013 to Feb 2017. During this period 1184 breast cancer patients received genetic testing because they fulfilled an eligibility criterion, 707 through oncology and 477 through genetics. The details of this dataset are provided at https://osf.io/twqfz/. BRCA results were communicated by letter; >90% within 3 weeks and 100% within 4 weeks. Mutation-positive individuals were sent a genetics clinic appointment with their result, together with a number to call should they have questions beforehand or a need to change the appointment time (Supplement eFigure 1).[1]

© 2019 Kemp Z et al. *JAMA Network Open*.

Cancer predisposition gene testing

Genetic testing was performed using the TruSight Cancer Panel in an ISO15189 accredited clinical testing laboratory, TGLclinical, as previously described.[4] We analysed nine breast cancer predisposition genes (*BRCA1*, *BRCA2*, *PALB2*, *TP53*, *PTEN*, *STK11*, *CDH1*, *ATM*, *CHEK2*) in line with UK recommendations.[5] We tested for both small variants and exon deletion/duplications.[4] Pathogenic mutations were independently verified using a second aliquot of DNA by Sanger sequencing or MLPA, as appropriate. Pathogenicity was determined in accordance with international guidelines and consistent with ClinVar designations.[6] If a variant was plausibly pathogenic and a specific evaluation could result in definitive classification, the variant was designated a Variant Requiring Evaluation (VRE) and the specific evaluation performed. We identified two VREs during the programme, *BRCA2* c.425G>A_p.Ser142Asn and *BRCA1* c.213-11T>G. Following evaluation both were classified and reported as pathogenic.

Patient and cancer team acceptability

We evaluated patient and cancer team acceptability of the mainstream process using similar questionnaires to the equivalent ovarian cancer programme.[1] We initiated the patient feedback once 250 patients had been consented by the cancer team, sending questionnaires to 259 participants, of whom 129 replied (113 BRCA mutation-negative and 16 BRCA mutation-positive). Not everyone answered every question and hence the denominator is not always 129. Twenty-three members of the cancer team completed the cancer team feedback questionnaire, including 12 oncologists, eight surgeons and three nurse specialists.

FH-series

Whilst the MCG-breast programme was running 182 breast cancer patients had genetic testing through genetics because although the proband did not fulfil an MCG-breast criterion, their family history (FH) made them eligible as they had a Manchester Score ≥15 (Supplement eFigure 2). These individuals are called the FH-series. Further details of this dataset are provided at https://osf.io/twqfz/.

BOCS analyses

We used data from the Breast and Ovarian Cancer Susceptibility (BOCS) study to evaluate the mutation detection rates of the MCG-breast criteria. BOCS is a national UK study aimed at identifying breast cancer predisposition genes and breast cancer patients were eligible if they had two or more relatives with breast cancer.[7] Participants were recruited to BOCS)study through genetic centres throughout the UK by 273 geneticists and genetic counsellors. For this study we included 2294 individuals with data on age at diagnosis, receptor status and number of primary

© 2019 Kemp Z et al. *JAMA Network Open*.

breast cancers. Individuals with ovarian cancer or male breast cancer were excluded from BOCS until 2012 and hence we were not able to use BOCS data to evaluate these eligibility criteria. Further details of this dataset are provided at https://osf.io/twqfz/. The research was approved by the London Multicentre Research Ethics Committee (MREC/01/2/18).

Malaysia-BCGS analyses

We used the data from the Malaysian Breast Cancer Genetic Study (BCGS), a prospective breast cancer case and control study from Malaysia, to evaluate the mutation detection rates using the MCG-breast criteria.[8] The study included 2575 patients with female breast cancer unselected for age or family history. Male breast cancer was not included so we could not use the data to evaluate this criterion. Only small variants were consistently detected, testing for exon deletion/duplications was not performed.[8]

Comparisons with other eligibility criteria

We compared MCG criteria with three widely-used systems for determining BRCA testing eligibility: the Manchester Scoring System (MSS1),[9] BOADICEA [10] and NCCN Guidelines Genetic/Familial High-Risk Assessment: Breast and Ovarian Version 2.2017.[3] We implemented MSS1 as shown in Supplement eFigure 2, using a cut-off of ≥15. To calculate BOADICEA values we generated lineage data with age, year of birth, cancer diagnosis and receptor status for all patients and relatives. If age was not known, we imputed values from the proband, assuming an average generation gap of 25 years. We used the BOADICEA Web Application v3 (http://ccge.medschl.cam.ac.uk/boadicea/) to generate the likelihood of a BRCA mutation being present, using a cut-off of ≥10, which is equivalent to a 10% chance of having a BRCA mutation.[10]

Cost-effectiveness analyses

We performed a cost-effectiveness analysis using the model in Eccleston et al, adapted for breast cancer.[11] A patient-level simulation with a lifetime time horizon was constructed in Microsoft Excel. In the model, an index population is compared for testing and no testing scenarios, patients are offered BRCA testing using the mainstream testing pathway and cascade testing to unaffected family members is included. The original model was for ovarian patients and we adapted it for breast cancer as follows: 1) Males with breast cancer were included in the index population. 2) Women with breast and ovarian cancer were excluded (as they are included in the ovarian cancer cost-effectiveness analysis). 3) Future risk of ovarian cancer was included for female members of the index population, assuming a breast cancer survival rate of 78% (http://www.cancerresearchuk.org/health-professional/cancer-statistics/statistics-by-cancer-type/breast-cancer/survival). 4) Breast cancer health utilities and age-adjusted death risks were applied to the index population. 5) A simplifying assumption excluding future risk of breast cancer

© 2019 Kemp Z et al. *JAMA Network Open*.

for the index population was applied. All other model parameters including cancer risks, survival rates, treatment costs and health utilities were as published.[11] The analysis was conducted according to the Consolidated Health Economic Evaluation Reporting Standards (CHEERS) guideline.

The index population numbers were based on the percentage of patients with breast cancer estimated to meet the MCG or MCGplus criteria from the MCG-breast and BOCS data and relevant publications.[12-15] applied to the UK breast cancer incidence data (http://www.cancerresearchuk.org/health-professional/cancer-statistics/statistics-by-cancer-type/breast-cancer). We applied a 0.8 overlap factor (number of patients/number of criteria met; 1184/1454) to correct for individuals meeting more than one criteria.

The model outputs were total and disaggregated costs and quality-adjusted life-years (QALYs), which were calculated for each individual and aggregated to provide an incremental cost-effectiveness ratio (ICER). The model also calculated the number of new cancer cases prevented and the number of lives saved over a 50 year time horizon. The perspective adopted was that of the National Healthcare Service (NHS) and personal social services, in line with methodological guidelines for Health Technology Assessment (HTA) submissions in the UK. Costs and outcomes were discounted at 3.5%, in line with current NICE guidance (http://publications.nice.org.uk/guide-to-the-methods-of-technology-appraisal-2013-pmg9). We explored model uncertainty through probabilistic sensitivity analysis containing 5,000 simulations, whereby all parameters were assigned distributions and varied jointly, as previously described [11]. We constructed multiple cost-effectiveness acceptability curves of the probability of BRCA testing being cost-effective at a willingness to pay threshold of £20,000 / QALY.[16]

Time and resource requirements

We used the data from this study to estimate the time, testing and genetic consultation requirements to implement the mainstream BRCA test access model with the MCG or MCGplus criteria. We used the data from UK cancer genetic national consultation days in 2013 and 2017 to estimate these for the traditional access model.[17] Wait time for a cancer patient to receive a genetics appointment is very variable in UK, ranging from one week to one year, with some centers having different wait times for different clinical scenarios. The median wait time for an appointment to discuss BRCA testing was 16 weeks. The BRCA test turnaround time was 3-12 weeks with a median of 8 weeks.

© 2019 Kemp Z et al. *JAMA Network Open*.

References

1. George, A., Riddell, D., Seal, S., Talukdar, S., Mahamdallie, S., Ruark, E., Cloke, V. *et al.* Implementing rapid, robust, cost-effective, patient-centred, routine genetic testing in ovarian cancer patients. *Sci Rep* **6**, 29506 (2016).
2. National Collaborating Centre for, C. National Institute for Health and Clinical Excellence: Guidance. in *Familial Breast Cancer: Classification and Care of People at Risk of Familial Breast Cancer and Management of Breast Cancer and Related Risks in People with a Family History of Breast Cancer* (National Collaborating Centre for Cancer (UK) National Collaborating Centre for Cancer., Cardiff (UK), 2013).
3. Daly, M.B., Pilarski, R., Berry, M., Buys, S.S., Farmer, M., Friedman, S., Garber, J.E. *et al.* NCCN Guidelines Insights: Genetic/Familial High-Risk Assessment: Breast and Ovarian, Version 2.2017. *J Natl Compr Canc Netw* **15**, 9-20 (2017).
4. Mahamdallie, S., Ruark, E., Holt, E., Poyastro-Pearson, E., Renwick, A., Strydom, A., Seal, S. *et al.* The ICR639 CPG NGS validation series: A resource to assess analytical sensitivity of cancer predisposition gene testing. *Wellcome Open Res* **3**, 68 (2018).
5. Taylor, A., Brady, A.F., Frayling, I.M., Hanson, H., Tischkowitz, M., Turnbull, C. & Side, L. Consensus for genes to be included on cancer panel tests offered by UK genetics services: guidelines of the UK Cancer Genetics Group. *J Med Genet* **55**, 372-377 (2018).
6. Landrum, M.J. & Kattman, B.L. ClinVar at five years: Delivering on the promise. *Hum Mutat* **39**, 1623-1630 (2018).
7. Ruark, E., Snape, K., Humburg, P., Loveday, C., Bajrami, I., Brough, R., Rodrigues, D.N. *et al.* Mosaic PPM1D mutations are associated with predisposition to breast and ovarian cancer. *Nature* **493**, 406-10 (2013).
8. Wen, W.X., Allen, J., Lai, K.N., Mariapun, S., Hasan, S.N., Ng, P.S., Lee, D.S. *et al.* Inherited mutations in BRCA1 and BRCA2 in an unselected multiethnic cohort of Asian patients with breast cancer and healthy controls from Malaysia. *J Med Genet* **55**, 97-103 (2018).
9. Evans, D.G., Lalloo, F., Wallace, A. & Rahman, N. Update on the Manchester Scoring System for BRCA1 and BRCA2 testing. *J Med Genet* **42**, e39 (2005).
10. Lee, A.J., Cunningham, A.P., Kuchenbaecker, K.B., Mavaddat, N., Easton, D.F. & Antoniou, A.C. BOADICEA breast cancer risk prediction model: updates to cancer incidences, tumour pathology and web interface. *Br J Cancer* **110**, 535-45 (2014).
11. Eccleston, A., Bentley, A., Dyer, M., Strydom, A., Vereecken, W., George, A. & Rahman, N. A Cost-Effectiveness Evaluation of Germline BRCA1 and BRCA2 Testing in UK Women with Ovarian Cancer. *Value Health* **20**, 567-576 (2017).
12. Dawood, S. Triple-negative breast cancer: epidemiology and management options. *Drugs* **70**, 2247-58 (2010).
13. Hartman, M., Czene, K., Reilly, M., Adolfsson, J., Bergh, J., Adami, H.O., Dickman, P.W. *et al.* Incidence and prognosis of synchronous and metachronous bilateral breast cancer. *J Clin Oncol* **25**, 4210-6 (2007).
14. Mellemkjaer, L., Friis, S., Olsen, J.H., Scelo, G., Hemminki, K., Tracey, E., Andersen, A. *et al.* Risk of second cancer among women with breast cancer. *Int J Cancer* **118**, 2285-92 (2006).
15. Pal, S., Luchtenborg, M., Davies, E.A. & Jack, R.H. The treatment and survival of patients with triple negative breast cancer in a London population. *Springerplus* **3**, 553 (2014).
16. Fenwick E, Byford S. A guide to cost-effectiveness acceptability curves. Br J Psychiatry **187**, 106-8 (2005)
17. Slade, I., Riddell, D., Turnbull, C., Hanson, H. & Rahman, N. Development of cancer genetic services in the UK: A national consultation. *Genome Med* **7**, 18 (2015).

eTable 1. BRCA mutation-positive individuals identified in the MCG-breast study

ID	MCG-breast category	Gene	Mutation	MSS1	BOADICEA	NCCN eligible
267851	BC, ≤45yrs	BRCA1	Exon 24 deletion	24	3.3	Y
292408	BC, ≤45yrs	BRCA1	c.4065_4068delTCAA	10	0.9	Y
312450	BC, ≤45yrs	BRCA2	c.517-2A>G	8	0.3	Y
358596	BC, ≤45yrs	BRCA2	c.9382C>T_p.Arg3128X	49	66.4	Y
366237	BC, ≤45yrs	BRCA2	c.5682C>G_p.Tyr1894X	20	3.8	Y
367643	BC, ≤45yrs	BRCA2	c.755_758delACAG	26	95.7	Y
376340	BC, ≤45yrs	BRCA2	c.4478_4481delAAAG	8	0.9	Y
507989	BC, ≤45yrs	BRCA1	Exon 21-24 deletion	33	84.4	Y
514692	BC, ≤45yrs	BRCA2	c.5946delT	18	2.2	Y
551858	BC, ≤45yrs	BRCA2	c.8933C>A_p.Ser2978X	16	2.8	Y
571707	BC, ≤45yrs	BRCA2	c.6835_6836insCTTTGTGGTAAGT	10	6.7	Y
573381	BC, ≤45yrs	BRCA2	c.7436-1G>T	27	9.3	Y
585911	BC, ≤45yrs	BRCA2	c.3717delA	13	1	Y
590830	BC, ≤45yrs	BRCA2	Exon 3 deletion	16	4.4	Y
609506	BC, ≤45yrs	BRCA2	c.8954-1G>A	10	1.1	Y
614576	BC, ≤45yrs	BRCA2	c.475G>C_p.Val159Leu	12	3.8	Y
621587	BC, ≤45yrs	BRCA1	c.5335C>T_p.Gln1779X	12	1.1	Y
623731	BC, ≤45yrs	BRCA2	c.8756delG	33	7.6	Y
623866	BC, ≤45yrs	BRCA2	c.7575delA	8	1	Y
636203	BC, ≤45yrs	BRCA2	c.67+1G>A	16	1.7	Y
636533	BC, ≤45yrs	BRCA2	c.8537_8538delAG	30	6.2	Y
641509	BC, ≤45yrs	BRCA2	c.5286T>G_p.Tyr1762X	10	4.4	Y
644094	BC, ≤45yrs	BRCA1	c.2475delC	18	4.7	Y
644118	BC, ≤45yrs	BRCA1	c.5057A>G_p.His1686Arg	8	2.6	Y
645603	BC, ≤45yrs	BRCA2	c.8575delC	16	12.3	Y
652150	BC, ≤45yrs	BRCA2	c.8329A>T_p.Lys2777X	13	4.1	Y
656801	BC, ≤45yrs	BRCA2	c.8660C>G_p.Ser2887X	10	6.9	Y
661442	BC, ≤45yrs	BRCA2	c.1555delA	11	5	Y
662222	BC, ≤45yrs	BRCA2	c.2808_2811delACAA	12	2.6	Y
662629	BC, ≤45yrs	BRCA2	c.4461dupA	21	9.5	Y
664521	BC, ≤45yrs	BRCA2	c.9154C>T_p.Arg3052Trp	8	1.9	Y
667960	BC, ≤45yrs	BRCA1	c.2766delA	6	0.8	Y
672030	BC, ≤45yrs	BRCA2	c.5119dupA	14	4.3	Y
672385	BC, ≤45yrs	BRCA1	c.5117G>A_p.Gly1706Glu	8	1.2	Y
673198	BC, ≤45yrs	BRCA2	c.1310_1313delAAGA	21	3.9	Y
678130	BC, ≤45yrs	BRCA2	c.5946delT	11	2.1	Y
680500	BC, ≤45yrs	BRCA2	c.5946delT	9	4.7	Y
574398	BC+OC	BRCA1	c.68_69delAG	28	57.2	Y
577235	BC+OC	BRCA1	c.2475delC	32	52.6	Y
586352	BC+OC	BRCA2	c.793+1G>A	19	27.1	Y
626714	BC+OC	BRCA1	c.5503C>T_p.Arg1835X	14	3.4	Y
632367	BC+OC	BRCA1	c.895_896delGT	21	35.5	Y
641903	BC+OC	BRCA2	c.3847_3848delGT	23	54.9	Y
647097	BC+OC	BRCA2	c.2330dupA	16	4.6	Y
658756	BC+OC	BRCA1	c.3331_3334delCAAG	26	0	Y
670407	BC+OC	BRCA1	c.68_69delAG	35	92.9	Y
585576	Male BC	BRCA2	c.8878C>T_p.Gln2960X	22	88.9	Y
648329	Male BC	BRCA2	c.8575delC	12	0.3	Y
665364	Male BC	BRCA2	c.6577G>T_p.Glu2193X	11	2.8	Y

© 2019 Kemp Z et al. *JAMA Network Open*.

ID	MCG-breast category	Gene	Mutation	MSS1	BOADICEA	NCCN eligible
284289	TN BC	BRCA1	c.68_69delAG	2	0.6	N
340732	TN BC	BRCA1	c.2188G>T_p.Glu730X	2	2.4	N
374870	TN BC	BRCA2	c.5073dupA	16	2.6	Y
381684	TN BC	BRCA1	c.68_69delAG	14	3.8	Y
390078	TN BC	BRCA1	c.4391delC	16	8.3	Y
501284	TN BC	BRCA1	c.2612_2613insT	8	1.7	Y
516570	TN BC	BRCA1	c.3833delA	6	0.2	Y
538945	TN BC	BRCA2	c.67+3A>G	16	16.5	Y
548488	TN BC	BRCA1	c.5266dupC	18	14	Y
565005	TN BC	BRCA2	c.5350_5351delAA	16	0.5	Y
589329	TN BC	BRCA2	c.4876_4877delAA	2	0.7	N
622958	TN BC	BRCA1	c.4327C>T_p.Arg1443X	26	5.1	Y
626746	TN BC	BRCA1	c.5266dupC	21	29.6	Y
627909	TN BC	BRCA2	c.1325C>A_p.Ser442X	18	1.3	Y
628771	TN BC	BRCA1	c.4327C>T_p.Arg1443X	8	0.6	Y
631683	TN BC	BRCA1	c.4484G>T_p.Arg1495Met	18	40.7	Y
634833	TN BC	BRCA2	c.658_659delGT	16	9	Y
638031	TN BC	BRCA1	c.181T>G_p.Cys61Gly	12	4.5	Y
640438	TN BC	BRCA1	Exon 13 duplication	24	24	Y
644385	TN BC	BRCA2	c.5946delT	4	1.3	Y
645691	TN BC	BRCA1	c.68_69delAG	8	1.7	Y
646593	TN BC	BRCA1	c.2681_2682delAA	10	10.4	Y
647862	TN BC	BRCA1	c.2241delC	16	4.9	Y
650573	TN BC	BRCA1	c.5266dupC	8	4.4	Y
652160	TN BC	BRCA1	Exon 22 deletion	33	85.4	Y
653661	TN BC	BRCA1	c.3331_3334delCAAG	20	30.3	Y
653911	TN BC	BRCA1	c.5058T>A_p.His1686Gln	29	40.6	Y
658335	TN BC	BRCA1	c.2681_2682delAA	17	42.7	Y
660776	TN BC	BRCA1	Exon 14-20 deletion	7	2.7	Y
663746	TN BC	BRCA1	c.4136_4137delCT	11	1.9	Y
668685	TN BC	BRCA1	Exon 24 deletion	16	2.4	Y
671381	TN BC	BRCA2	c.658_659delGT	4	1.9	N
672888	TN BC	BRCA2	c.5722_5723delCT	23	4.5	Y
675221	TN BC	BRCA1	c.1953_1956delGAAA	12	9.7	Y
681361	TN BC	BRCA1	c.3143delG	14	0.4	Y
682090	TN BC	BRCA1	c.212+3A>G	8	2.3	Y
204574	Two BCs ≤60yrs	BRCA2	c.2808_2811delACAA	16	1.4	Y
226578	Two BCs ≤60yrs	BRCA1	c.68_69delAG	16	7.7	Y
269539	Two BCs ≤60yrs	BRCA1	c.4542delT	14	1.5	Y
332417	Two BCs ≤60yrs	BRCA2	c.6275_6276delTT	13	5.2	Y
514378	Two BCs ≤60yrs	BRCA2	c.2808_2811delACAA	8	1	N
516838	Two BCs ≤60yrs	BRCA1	c.1961delA	25	28.5	Y
539003	Two BCs ≤60yrs	BRCA2	c.7988A>T_p.Glu2663Val	8	0.1	Y
539258	Two BCs ≤60yrs	BRCA2	c.7988A>T_p.Glu2663Val	18	16	Y
562556	Two BCs ≤60yrs	BRCA2	Exon 14-16 deletion	28	48.1	Y
574764	Two BCs ≤60yrs	BRCA1	c.68_69delAG	31	58.2	Y
585325	Two BCs ≤60yrs	BRCA2	c.5946delT	11	0.1	N
607006	Two BCs ≤60yrs	BRCA2	c.3922G>T_p.Glu1308X	33	63.6	Y
613600	Two BCs ≤60yrs	BRCA2	c.5073dupA	12	5.6	Y
630706	Two BCs ≤60yrs	BRCA1	c.427G>T_p.Glu143X	25	36.8	Y
637868	Two BCs ≤60yrs	BRCA1	Exon 20 deletion	16	16.7	Y

© 2019 Kemp Z et al. *JAMA Network Open*.

ID	MCG-breast category	Gene	Mutation	MSS1	BOADICEA	NCCN eligible
644838	Two BCs ≤60yrs	BRCA2	c.3371_3372insAA	12	0.9	Y
646100	Two BCs ≤60yrs	BRCA1	c.5503C>T_p.Arg1835X	14	46.7	Y
648350	Two BCs ≤60yrs	BRCA2	c.2330dupA	8	3.2	N
649775	Two BCs ≤60yrs	BRCA2	c.4111C>T_p.Gln1371X	14	0.2	Y
653156	Two BCs ≤60yrs	BRCA2	c.6275_6276delTT	12	4.4	Y
654906	Two BCs ≤60yrs	BRCA2	c.2808_2811delACAA	14	11.1	Y
680990	Two BCs ≤60yrs	BRCA2	Exon 8 deletion	12	10.4	N
684285	Two BCs ≤60yrs	BRCA1	c.5444G>A_p.Trp1815X	8	1.1	N
619554	Two BCs ≤60yrs+OC	BRCA1	c.1175_1214del40	38	99.1	Y
271660	Two BCs ≤60yrs+TN BC	BRCA1	c.3331_3334delCAAG	14	7.2	Y
381948	Two BCs ≤60yrs+TN BC	BRCA1	c.2389_2390delGA	10	0.1	N
385613	Two BCs ≤60yrs+TN BC	BRCA1	c.3254_3255dupGA	4	6.2	N
525574	Two BCs ≤60yrs+TN BC	BRCA1	Exon 18-19 deletion	14	12.4	Y
532291	Two BCs ≤60yrs+TN BC	BRCA1	c.68_69delAG	15	37.3	Y
624143	Two BCs ≤60yrs+TN BC	BRCA1	Exon 21-24 deletion	16	22.5	Y
625779	Two BCs ≤60yrs+TN BC	BRCA2	c.7934delG	36	83.1	Y
638649	Two BCs ≤60yrs+TN BC	BRCA1	c.213-11T>G	14	12.6	Y

eTable 2. Summary of patient feedback

Question		No mutation	Mutation	Totals	Total %
I was given a Patient Information Sheet prior to my test	Agree	101	12	**113**	**88%**
	Unsure	8	4	**12**	**9%**
	Disagree	3	0	**3**	**2%**
The written information provided was clear and helpful	Agree	101	13	**114**	**90%**
	Unsure	8	3	**11**	**9%**
	Disagree	2	0	**2**	**2%**
I was clear in my own mind why I was being offered a genetic test.	Agree	110	16	**126**	**98%**
	Unsure	2	0	**2**	**2%**
	Disagree	0	0	**0**	**0%**
I was given sufficient time to think about whether I wanted a test	Agree	107	15	**122**	**95%**
	Unsure	3	0	**3**	**2%**
	Disagree	2	1	**3**	**2%**
I was aware that I could have additional discussions with the Genetics team before deciding whether to have a test.	Agree	74	13	**87**	**68%**
	Unsure	26	3	**29**	**23%**
	Disagree	12	0	**12**	**9%**
I was aware that the result of the test might impact me.	Agree	109	16	**125**	**98%**
	Unsure	3	0	**3**	**2%**
	Disagree	0	0	**0**	**0%**
I was aware that the result of the test might have implications for my family.	Agree	113	16	**129**	**100%**
	Unsure	0	0	**0**	**0%**
	Disagree	0	0	**0**	**0%**
I was aware that further discussions with the Genetics team would be organised for me if a gene mutation was found.	Agree	95	15	**110**	**85%**
	Unsure	16	1	**17**	**13%**
	Disagree	2	0	**2**	**2%**
I was informed when the result would be available.	Agree	96	15	**111**	**86%**
	Unsure	13	0	**13**	**10%**
	Disagree	4	1	**5**	**4%**
I was informed how I would receive the result.	Agree	87	12	**99**	**77%**
	Unsure	18	3	**21**	**16%**
	Disagree	8	1	**9**	**7%**
When I received my result I was given a Patient Information Sheet about receiving a a BRCA1 and BRCA2 test result that identifies a normal BRCA1 and BRCA2 result.	Agree	92		**92**	**84%**
	Unsure	14		**14**	**13%**
	Disagree	4		**4**	**4%**
When I received my result, I also received a Patient Information Sheet about receiving a BRCA1 and BRCA2 test result that identifies a mutation.	Agree		15	**15**	**94%**
	Unsure		1	**1**	**6%**
	Disagree		0	**0**	**0%**
I was happy to receive my results by letter.	Agree	100	12	**112**	**90%**
	Unsure	4	1	**5**	**4%**
	Disagree	6	1	**7**	**6%**
I was happy to receive a copy of my test report (results).	Agree	103	13	**116**	**93%**
	Unsure	7	0	**7**	**6%**
	Disagree	2	0	**2**	**2%**
I was happy to have the genetic test at one of my existing Oncology appointments rather than in a separate appointment with the Genetics team.	Agree	108	16	**124**	**96%**
	Unsure	4	0	**4**	**3%**
	Disagree	1	0	**1**	**1%**
I am pleased I had the genetic test.	Agree	113	15	**128**	**100%**
	Unsure	0	0	**0**	**0%**
	Disagree	0	0	**0**	**0%**
I understood the implications of this result for me.	Agree	107		**107**	**95%**
	Unsure	6		**6**	**5%**
	Disagree	0		**0**	**0%**
When I received my result, I also received an appointment with the Genetics Team.	Agree		14	**14**	**88%**
	Unsure		2	**2**	**13%**
	Disagree		0	**0**	**0%**
My appointment with the Genetics team helped me to understand what the result meant for me and for my family.	Agree		16	**16**	**100%**
	Unsure		0	**0**	**0%**
	Disagree		0	**0**	**0%**

Note: Not every respondee answered every Q - hence question summary totals <129).

© 2019 Kemp Z et al. *JAMA Network Open.*

eTable 3. Summary of cancer team feedback

Question	Average Score
I believe it is important for breast cancer patients to be able to benefit from BRCA gene testing.	4.7
There is increasing interest from breast cancer patients to have BRCA gene testing.	4.3
I welcome the opportunity to carry out BRCA gene testing for breast cancer patients through Breast Unit appointments.	4.7
I found it helpful to have supporting materials (e.g. training materials and FAQs) containing information on carrying out BRCA gene testing.	4.7
It was useful to be able to complete the BRCA gene testing training materials at a time that was convenient for me.	4.7
It is useful to have an approved clinical protocol to follow when carrying out BRCA gene testing.	4.0
It is useful to have information sheets to provide to patients about BRCA gene testing.	4.8
I feel confident to consent a patient for a BRCA gene test.	4.3
It is possible to discuss BRCA gene testing with a patient within the timeframe of a consultation.	4.0
I was clear when the BRCA gene test result would be available.	4.3
The process for carrying out BRCA gene testing worked well.	4.6

Responses were scored as follows:
1=Strongly disagree 2=Disagree 3=Unsure 4=Agree 5=Strongly agree

eTable 4. Eligibility and family history of BRCA mutation-positive individuals in FH-series

ID	Cancer in proband, Age (yrs)	FDR eligible by MCG criteria	MCG eligible cancer in relatives with age + relationship to proband	Eligible relative diagnosed before proband	Manchester Score	BOADICEA ≥10	NCNN all criteria	NCNN personal cancer criteria
351676	BC 47	Y	BC 44 (FDR), BC 33 (SDR)	Y	22	N	Y	N
439596	BC 55	U	bilateral BC 36 (TDR)	Y	26	N	Y	N
533429	BC 52	Y	OC 50 (FDR)	N	17	N	Y	N
555132	Two primary BCs 59+66	Y	OC (FDR), BC 40 (FDR), BC 42 (FDR)	Y	40	N	Y	N
580376	BC 48	U	BC (FDR), BC+OC (SDR)	Y	18	N	Y	N
589102	BC 65	Y	BC 37 (FDR), BC 35 (SDR)	Y	34	N	Y	N
622590	Two primary BCs 53+74	Y	BC 34 (FDR), BC 45 (SDR)	Y	19	Y	Y	N
629322	BC 63	Y	OC 39 (FDR)	Y	15	N	Y	N
636095	BC 58	N	OC (SDR), BC 37 (SDR), BC 40 (SDR), BC+OC 55 (TDR)	Y	49	Y	Y	N
653420	BC 60	N	OC 44 (SDR)	Y	21	N	Y	N

Notes: FDR, first-degree relative; BC, breast cancer; OC, ovarian cancer; SDR, second-degree relative; TDR, third-degree relative;

© 2019 Kemp Z et al. *JAMA Network Open*.

eTable 5. Mutations in non-BRCA breast cancer predisposition genes

Patient Group	No. of mutations	% with mutations
**MCG-breast (n=1184)**		
PALB2	15	1.7
PTEN	1	0.08
STK11	1	0.08
TP53	6	0.5
CDH1	1	0.08
All higher penetrance genes	24	2
ATM	11	0.9
CHEK2	15	1.7
ATM+CHEK2	1	0.08
All lower penetrance genes	27	2.2
**All genes**	**51**	**4.3**
**FH-series (n=182)**		
PALB2	1	0.05
All higher penetrance	1	0.05
ATM	3	1.6
CHEK2	5	2.7
All lower penetrance genes	8	4.4
**All genes**	**9**	**4.9**

© 2019 Kemp Z et al. *JAMA Network Open.*

eTable 6. Annual BRCA tests required in UK using MCG or MCGplus criteria

Criteria	Number/year (UK)
1. Ovarian cancer	7270
2. BC, ≤45yrs	4986
3. Two primary BCs, both ≤60yrs	1022
4. Triple negative BC	8268
5. Male BC	371
6. BC + parent, child or sibling with any of the above criteria	4947
**MCG criteria total (1-5)***	**19476**
**MCGplus criteria total (1-6)***	**24422**

*0.8 overlap factor applied to correct for patients meeting multiple criteria

© 2019 Kemp Z et al. *JAMA Network Open*.

eFigure 1. MCG-breast process and patient flow timeline

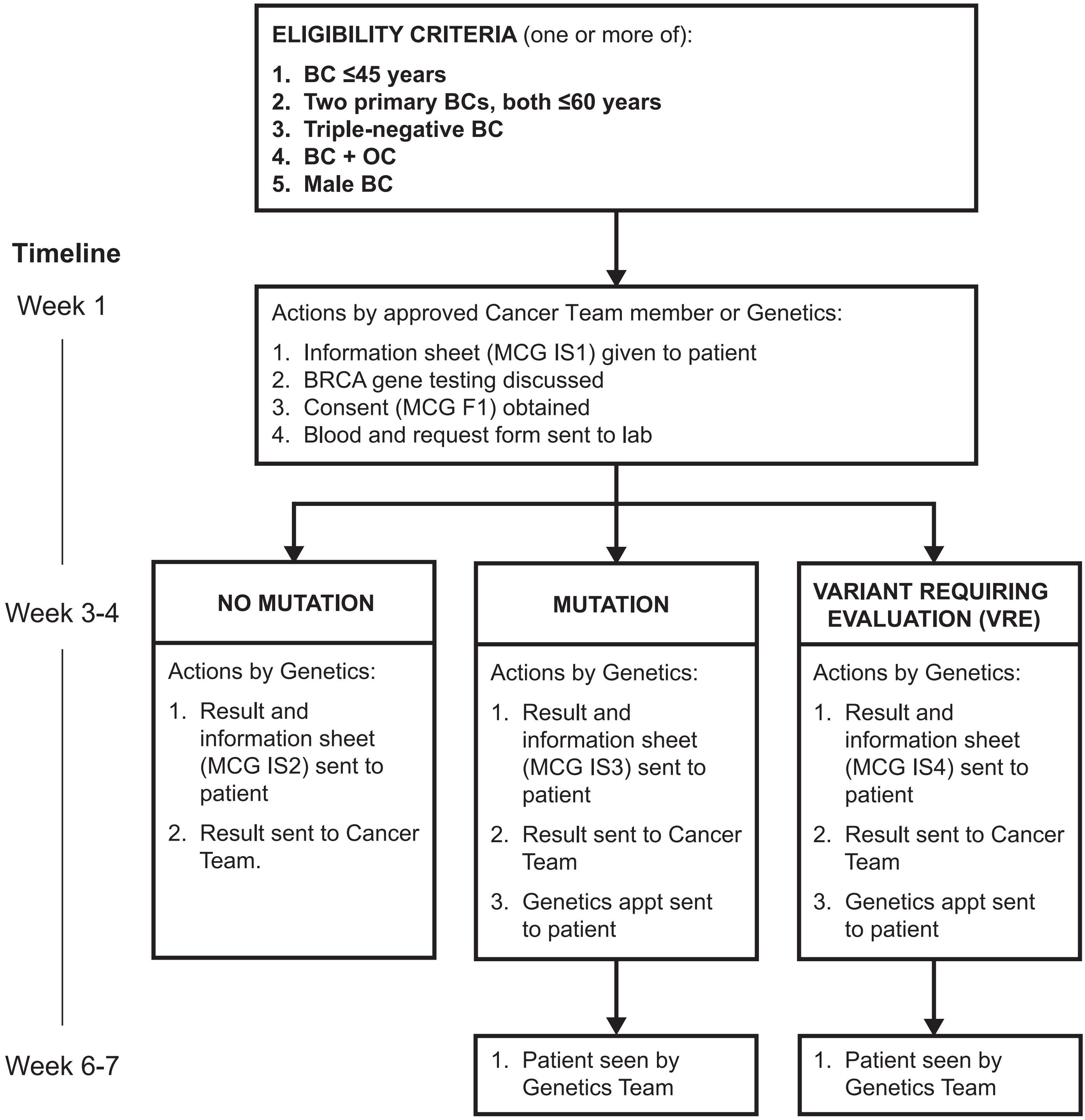

Notes: BC = Breast cancer, OC = Epithelial Ovarian cancer. Further details are given in the Supplementary eProtocol

© 2019 Kemp Z et al. *JAMA Network Open*.

eFigure 2. BRCA testing protocol used in genetics prior to MCG-breast programme

ICR

The Royal Marsden NHS
NHS Foundation Trust

Protocol 2
BRCA1 and *BRCA2* mutation testing guidelines[1]

A
Woman with **breast cancer** who →

1) has bilateral BC and both cancers diagnosed < 50 yrs
2) has triple negative BC diagnosed < 50 yrs
3) has OC
4) has bilateral BC and a relative with BC < 60 yrs
5) has a relative with BC and both diagnosed < 45 yrs
6) has relatives with cancer and a Manchester Score ≥15

B
Woman with **ovarian cancer** who →

1) Has non-mucinous OC diagnosed at any age
2) has another primary cancer diagnosis[2]
3) has a relative with OC or MBC
4) has relatives with cancer and a Manchester Score ≥ 15

C
Male with **breast cancer** who →

1) has a relative with OC or MBC
2) has relatives with cancer and a Manchester Score ≥ 15

D
Individual **unaffected** with cancer who →

1) has relatives with breast and/or ovarian cancer and a Manchester Score ≥ 17 (see FAQ for full eligibility)

Results
***BRCA1/2* mutation identified:** see Protocol 3
***BRCA1/2* mutation not identified:** see Protocol 1 for breast screening recommendation
***BRCA1 /2* variant identified:** TGL report will be issued for internal results. For external results email: vus@icr.ac.uk

Notes
[1] The above criteria relate to a full gene screen of *BRCA1* and *BRCA2*, individuals of Ashkenazi Jewish heritage may be eligible for founder mutation testing.*
[2] Includes individuals with mucinous ovarian cancer and another primary cancer diagnosis

Key
Relative = first degree or second degree relative only, except when calculating a Manchester score*. Female relatives through an intervening male shift up one degree of relationship*
BC = breast cancer **MBC** = male breast cancer **OC** = ovarian cancer
Triple negative breast cancer = breast tumour negative for oestrogen receptor (ER), progesterone receptor (PR) and HER2 Expression
TGL=TGL clinical laboratory

Manchester Score*

Cancer, age at diagnosis	Score
♀ Breast Cancer, <30	11
♀ Breast Cancer, 30-39	8
♀ Breast Cancer, 40-49	6
♀ Breast Cancer, 50-59	4
♀ Breast Cancer, > 59	2
♂ Breast Cancer, <60	13
♂ Breast Cancer, > 59	10
Ovarian Cancer, <60	13
Ovarian Cancer, >59	10
Pancreatic Cancer	1
Prostate Cancer, <60	2
Prostate Cancer, >59	1

* *See FAQ document. http://www.icr.ac.uk/protocols* 14/05/2014

© 2019 Kemp Z et al. *JAMA Network Open*.

eFigure 3. Impact on testing and genetic consultations requirements of implementing MCGplus in UK

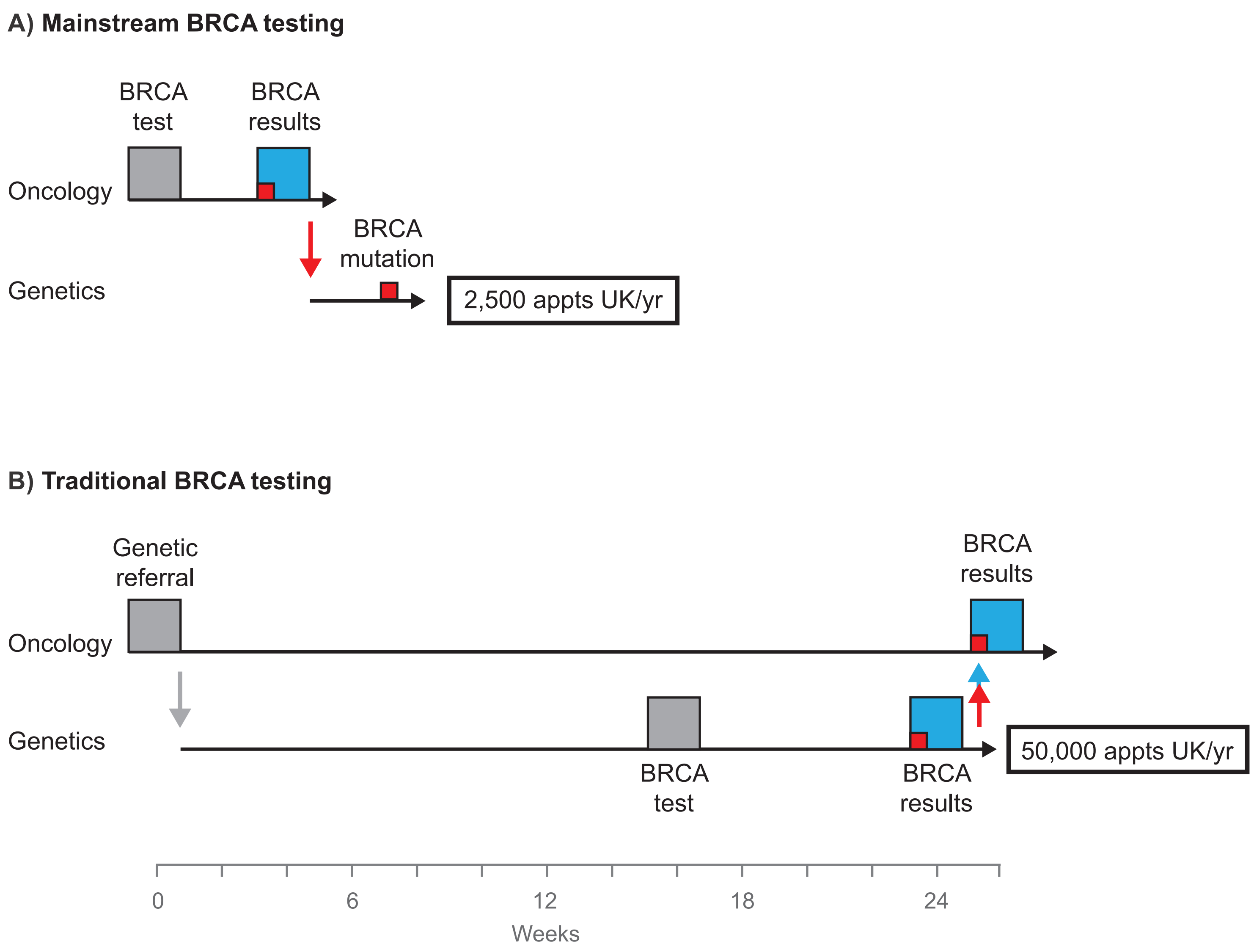

eFigure 3. A) In the mainstream access model BRCA status is known and can be used for cancer management within four weeks. The 10% of individuals with mutations have genetic consultations (in red). In the UK this would be 2,500 genetic consultations if MCGplus criteria are used. B) In the traditional testing model all patients have genetic input before and after testing. In the UK the average wait time for a genetic appointment is 16 weeks, and the average test turnaround time is 8 weeks. Thus the BRCA status is not known for ~25 weeks and 50,000 genetic consultations would be required if MCGplus criteria are used. Grey box, untested individuals; red box, individuals with BRCA mutation; blue box, individuals without BRCA mutation.

© 2019 Kemp Z et al. *JAMA Network Open*.

eFigure 4. BRCA testing protocol used in oncology and genetics from 11/2017

The ROYAL MARSDEN
NHS Foundation Trust

Protocol 2
BRCA1 and BRCA2 mutation testing

Eligibility criteria

Individual with any of the following:

1) Ovarian cancer
2) Breast cancer ≤45 years
3) Two primary breast cancers, both ≤60 years
4) Triple-negative breast cancer
5) Male breast cancer
6) Breast cancer + parent, child or sibling with any of the above criteria

Individuals **not** meeting any of the eligibility criteria can have a self-funded test.

Process

Actions by Oncology or Genetics:

1. Information sheet (MCG IS1) given to patient.
2. BRCA gene testing discussed.
3. Consent (MCG F1) obtained.
4. Blood and test request form sent to lab.

NO MUTATION

Actions by Genetics:

1. Result and information sheet (MCG IS2) sent to patient.
2. Result sent to Cancer Team.

MUTATION

Actions by Genetics:

1. Result and information sheet (MCG IS3) sent to patient.
2. Result sent to Cancer Team.
3. Genetics appt sent to patient.

VARIANT REQUIRING EVALUATION (VRE)

Actions by Genetics:

1. Result and information sheet (MCG IS4) sent to patient.
2. Result sent to Cancer Team.
3. Genetics appt sent to patient.

See Protocol 2 FAQ

© 2019 Kemp Z et al. *JAMA Network Open*.

04/11/2017 v7
